# Mineral Composition and Adequacy of Mineral Concentrations in Dog and Cat Diets: A Systematic Review

**DOI:** 10.3390/ani16203261

**Published:** 2026-10-16

**Authors:** Marta López-Alonso, Belén Larrán, Nekane Flores, Inmaculada Orjales, Marta Miranda

**Affiliations:** 1 Institute for Research in Global Health and Sustainable Development, iTERRA, Department of Animal Pathology, Faculty of Veterinary Medicine, Universidade de Santiago de Compostela, Campus Terra, 27002 Lugo, Spain; belen.larran.franco@usc.es; 2 Faculty of Veterinary Medicine, Universidade de Santiago de Compostela, Campus Terra, 27002 Lugo, Spain; nekane.flores@rai.usc.es; 3 Institute for Research in Global Health and Sustainable Development, iTERRA, Department of Anatomy, Animal Production and Veterinary Clinical Sciences, Faculty of Veterinary Medicine, Universidade de Santiago de Compostela, Campus Terra, 27002 Lugo, Spain; inma.orjales@usc.es (I.O.); marta.miranda@usc.es (M.M.)

**Keywords:** pet food, animal nutrition, trace elements, microminerals, macrominerals

## Abstract

Minerals are essential for numerous physiological functions in dogs and cats; however, concerns remain regarding the adequacy and safety of mineral supply across an increasingly diverse range of diets. This systematic review synthesised evidence on mineral composition and adequacy in companion-animal diets, with emphasis on dietary factors influencing mineral variability. A systematic search identified studies published between 2005 and 2025 that evaluated mineral composition, intake or adequacy in conventional commercial, raw-meat-based, plant-based, home-prepared, and therapeutic diets. Seventy-six studies met the inclusion criteria, including 58 analytical studies and 18 studies based on estimated mineral intake or adequacy of mineral levels. Calcium and phosphorus were the most frequently reported macrominerals, and iron, zinc, copper, iodine, and selenium were the most commonly evaluated trace elements. Deficient and excessive mineral levels occurred across dietary categories, with substantial variability in several alternative-diet datasets and some conventional commercial diets. No uniform directional difference in mean mineral concentrations distinguished conventional from alternative diets, while price and commercial positioning did not consistently predict adequacy. Intake-based studies showed that feeding level can alter adequacy despite apparently adequate dietary concentrations. Methodological heterogeneity, limited bioavailability data, and inconsistent toxicological benchmarks constrain interpretation and highlight the need for more standardised assessment approaches.

## Simple Summary

Minerals are essential for the healthy development and functioning of dogs and cats, but both insufficient and excessive amounts may cause health problems. Pet feeding practices are becoming increasingly diverse and now include raw-meat-based, home-prepared, and plant-based diets, as well as conventional commercial foods. We reviewed 76 studies that examined the mineral content or estimated the amounts of minerals such diets provide. Mineral composition varied greatly among products and type of diet. Raw-meat-based, home-prepared, and plant-based diets frequently showed substantial variability in mineral composition, although considerable variability was also observed in some conventional commercial foods. However, potentially inadequate or excessive mineral levels were reported in all dietary categories examined, with recurrent multielement imbalances in several alternative-diet datasets. Price or premium positioning did not reliably indicate better mineral balance. Studies of potentially harmful elements also showed considerable variability and used different safety thresholds. Evidence remained limited regarding how much of the minerals present in diets are absorbed and used by animals and the effects of long-term exposure. Better monitoring of pet foods and more consistent research methods are needed to support well-informed feeding decisions.

## 1. Introduction

Adequate nutrition is a cornerstone of health, welfare, and longevity in companion animals [1]. In dogs and cats, minerals play essential roles in a wide range of physiological processes, including skeletal development, neuromuscular function, energy metabolism, endocrine regulation, and immune competence [2,3]. Macrominerals such as calcium, phosphorus, sodium, and potassium are fundamental for structural integrity, skeletal development, and electrolyte balance. Essential trace elements (microminerals), such as zinc, copper, iron, selenium, and iodine, are critical for maintaining metabolic homeostasis, even though they are only required in small amounts [4]. Conversely, chronic excessive intake of certain essential minerals, in addition to exposure to non-essential or potentially toxic elements, may contribute to tissue accumulation and adverse health effects [5]. Importantly, mineral imbalances may remain subclinical for prolonged periods, and their detection and prevention may thus be especially challenging in long-lived companion animals. These considerations are particularly relevant in companion animals, which are often fed the same commercial diet for prolonged periods due to strong caregiver loyalty to specific brands [6], making long-term nutritional balance a critical determinant of health and wellbeing [7].

In recent decades, feeding practices for dogs and cats have undergone substantial changes, accompanied by a marked diversification of available diets [8]. In addition to conventional dry extruded foods, a wide variety of moist canned diets, raw-meat-based diets (RMBDs), home-prepared diets, and veterinary therapeutic diets designed for specific physiological or pathological conditions are used. These diets vary in dietary category and also in format (dry versus moist), ingredient composition, degree of processing, use of animal by-products or offal, and reliance on mineral premixes. At the same time, consumer-driven trends such as grain-free formulations (i.e., diets formulated without cereal grains), high-meat products, minimally processed diets, and plant-based formulations have further increased the heterogeneity of feeding patterns [7,9,10]. This growing complexity raises important questions regarding the consistency of mineral supply across diet types and the potential for long-term imbalances or exposure associated with specific formulation strategies.

The nutritional adequacy of pet foods is generally assessed relative to established guidelines such as those proposed by the National Research Council [4], the Association of American Feed Control Officials (AAFCO) [11], and the European Pet Food Industry Federation (FEDIAF) [12]. These frameworks provide essential reference values for dietary formulation, typically expressed on a feed-weight basis and/or relative to metabolizable energy. However, compliance with recommended concentrations does not necessarily ensure adequate or safe mineral intake under real-life feeding conditions [4,13]. Factors such as energy density, feeding rate, caloric restriction, bioavailability, ingredient matrix, and processing technology can greatly modify mineral intake and utilization [14]. In particular, energy restriction, common in weight-loss programmes, may lead to insufficient mineral intake even when diets comply with concentration-based recommendations [15]. Conversely, certain feeding patterns, particularly those associated with specific ingredient choices, processing practices or energy density, may increase chronic exposure to specific minerals or undesirable elements, despite apparent compliance with regulatory limits [2].

A growing body of literature has evaluated mineral composition and, less frequently, mineral adequacy or exposure to non-essential elements in commercial diets, RMBDs, and home-prepared diets for dogs and cats. However, the evidence remains fragmented and highly heterogeneous. Many studies focus on a single dietary category or a limited number of minerals, and study designs vary widely regarding whether they report analytical concentrations in finished diets, estimate intake under specific feeding regimens, or summarise compliance relative to guideline thresholds. Studies integrating the multiple dietary and feeding-related factors that may influence mineral composition and adequacy are relatively scarce. Those examining essential and non-essential elements within a unified analytical framework are even rarer. As a result, the factors driving mineral variability, inadequacy risk, and chronic exposure across dog and cat diets have not yet been systematically characterised.

The aim of the present systematic review was to synthesise the available evidence on mineral composition and adequacy of complete diets for dogs and cats, with particular emphasis on dietary and feeding-related factors potentially influencing mineral balance. Data on essential minerals were considered, along with (when available) data on non-essential or potentially toxic elements. By adopting an integrative approach anchored to widely used nutritional guidelines (AAFCO and FEDIAF), this review seeks to identify consistent patterns and highlight factors associated with mineral imbalance or altered mineral intake. It also seeks to provide a structured framework to support clinical, nutritional, and regulatory decision-making in companion animal nutrition.

## 2. Materials and Methods

### 2.1. Study Design

This study was conducted as a systematic review following the Preferred Reporting Items for Systematic Reviews and Meta-Analyses (PRISMA) guidelines [16]. A review protocol was developed a priori in order to identify, select, and synthesise studies reporting data on mineral composition, adequacy or exposure in dog and cat diets, but was not formally registered.

A systematic literature search was performed in major scientific databases (Scopus, PubMed, Web of Science and AGRIS) to identify relevant articles published between 1 January 2005 and 4 October 2025. Two complementary search components were used to maximise retrieval of both studies reporting mineral concentrations in diets and studies addressing mineral intake or nutritional adequacy. The search strategy combined terms related to species, diet, and mineral composition or mineral adequacy.

The species-related terms included “dog”, “dogs”, “canine”, “cat”, “cats”, and “feline”. Diet-related terms included “pet food”, “dog food”, “dogfood”, “cat food”, “diet”, “food”, “feeding”, “ration”, and “recipe”, together with combinations of terms describing dietary formats or categories (“commercial”, “complete”, “dry”, “wet”, “canned”, “raw”, “BARF”, “homemade”, “home-prepared”, “vegan”, “vegetarian”, “therapeutic”, and “prescription”) with “diet” or “food”. Terms related to weight-management feeding were also included (“weight loss”, “weight reduction”, “weight management”, “energy restriction”, and “caloric restriction”) to retrieve studies evaluating mineral supply under restricted energy intake. Mineral-related terms included general descriptors (“mineral”, “trace element”, “micromineral”, “macromineral”, “heavy metal”, “potentially toxic element”, “chemical composition”, and “elemental composition”) and individual elements (“calcium”, “phosphorus”, “magnesium”, “sodium”, “potassium”, “chloride”, “chlorine”, “iron”, “zinc”, “copper”, “iodine”, “selenium”, “manganese”, “cobalt”, “molybdenum”, “arsenic”, “cadmium”, “lead”, “mercury”, “chromium”, and “nickel”). These were combined, as appropriate, with terms describing the study outcomes, including “content”, “concentration”, “composition”, “level”, “analysis”, “adequacy”, “intake”, “requirement”, “deficiency”, and “deficiencies”. The syntax was adapted to the indexing and search-field requirements of each database. The complete database-specific search strategies used for PubMed, Scopus, Web of Science, and AGRIS are provided in the Supplementary File S1. In addition, the reference lists of selected articles and relevant reviews were screened to identify additional eligible studies.

### 2.2. Eligibility Criteria

Studies were considered eligible for inclusion if they (i) evaluated complete diets or complete feeding regimens intended for dogs and/or cats, including commercial diets, RMBDs, home-prepared diets or veterinary therapeutic diets; (ii) reported information on mineral composition, dietary mineral intake or dietary exposure as a substantive study objective; (iii) provided sufficient information to characterise the complete diet or feeding regimen evaluated; (iv) were available as full-length articles in peer-reviewed journals containing original data and sufficient methodological detail for assessment; and (v) were written in English or Spanish.

Studies were excluded if they (i) did not report any data related to mineral content, intake or adequacy; (ii) reported mineral concentrations only as background characterisation of diets used to investigate outcomes unrelated to mineral nutrition; (iii) were experimental mineral intervention or dose–response studies primarily designed to investigate mineral metabolism, bioavailability, or physiological responses to specific mineral sources or doses, rather than the composition, adequacy, intake, or dietary exposure associated with a complete feeding regimen; (iv) focused primarily on the development or validation of an analytical method using pet foods only as test matrices, without an independent objective of characterising mineral composition or adequacy of the diets; (v) reported mineral data only for individual ingredients without reconstruction or evaluation of complete diets; or (vi) were conference abstracts, editorials, commentaries, academic theses/dissertations, or reviews without original data.

Titles, abstracts, and full-text articles were screened independently by two reviewers (N.F. and B.L.). Eligibility decisions were compared after each screening stage, and any uncertainties regarding eligibility were resolved through discussion and consensus.

### 2.3. Classification of Studies According to Data Type

To ensure methodological consistency and to support a robust review, the studies were classified according to the nature of the mineral data reported:

1. Analytical mineral composition studies: Studies reporting mineral concentrations measured directly in complete diets using laboratory analytical methods. Mineral concentrations were expressed on a feed-weight basis (e.g., g or mg per 100 g of product) and/or relative to metabolizable energy (per 1000 kcal).
2. Estimated intake or adequacy studies: Studies in which mineral content or intake was estimated using ingredient composition tables, product labels, dietary reconstruction from owner questionnaires or nutritional software, without direct chemical analysis of the finished diet.

Only studies reporting analytically measured mineral concentrations in complete diets were included in the quantitative review, tabulation of mineral values and graphical representation. Studies based on estimated data were retained exclusively for qualitative review and discussion, where they contributed to the interpretation of potential risks of mineral imbalance associated with specific feeding practices.

A comprehensive summary of all studies reviewed, including the study design, species, dietary characteristics, and type of mineral data reported, is provided in Supplementary Table S1 [17,18,19,20,21,22,23,24,25,26,27,28,29,30,31,32,33,34,35,36,37,38,39,40,41,42,43,44,45,46,47,48,49,50,51,52,53,54,55,56,57,58,59,60,61,62,63,64,65,66,67,68,69,70,71,72,73,74] and Table S2 [75,76,77,78,79,80,81,82,83,84,85,86,87,88,89,90,91,92].

### 2.4. Data Extraction

The following information was extracted (when available) from each analytical study: target species (dog or cat), type of diet evaluated (conventional commercial, alternative or therapeutic), dietary format (dry or moist), life stage or physiological context, minerals analysed, original units and basis of expression, and reported mineral values (mean ± standard deviation [SD], median, range or individual diet values). To facilitate comparison across studies, numerical units were harmonised. For studies reporting mineral concentrations on a dry-matter basis, macrominerals were expressed as g/100 g DM, trace elements as mg/100 g DM, and selenium as µg/100 g DM. Studies reporting concentrations relative to energy intake were retained on an energy basis and expressed per 1000 kcal ME. No conversion between dry-matter and energy bases was performed when this would have required assumptions regarding moisture content or energy density. Values reported on a different basis were retained and explicitly identified. Where individual diet values were available, arithmetic means and SD were calculated for comparative tabulation. When studies reported ranges without sufficient information to calculate mean and SD, ranges were retained as reported. SDs were not estimated from ranges or graphical data. Where SD could be derived directly from a reported standard error of the mean (SEM) and sample size, it was calculated as SD = SEM × √n. Data extraction was performed by N.F. using a standardized data extraction table and subsequently verified by M.L.-A. Any uncertainties or discrepancies were resolved through discussion and consensus.

### 2.5. Methodological and Analytical Quality Assessment

Because no validated appraisal tool is currently available for studies evaluating mineral concentrations in pet foods, a study-specific methodological and analytical quality assessment was developed. The instrument was designed to characterise study features relevant to sampling procedures, diet characterisation, sample preparation, analytical methodology, analytical quality control, and reporting completeness. It was not intended to provide a formal estimate of risk of bias. The criteria were jointly defined by two authors (B.L. and M.L-A.) with expertise in mineral analysis. The studies were independently evaluated by these authors, and assessments were reached by consensus. The complete assessment tool is presented in Table 1.

The assessment was applied exclusively to studies reporting analytically measured mineral concentrations in complete diets. Studies based on estimated mineral composition were not evaluated using this tool because estimated values reflect formulated or declared nutrient composition rather than the actual mineral concentrations in the final product. Therefore, the analytical quality domains included in the assessment were not applicable to these studies.

The assessment comprised 11 binary items (c1–c11; Table 1), each scored as 1 when the criterion was fulfilled and 0 when the criterion was not reported or not fulfilled. Total scores were calculated as the number of criteria fulfilled. For analytical methods that did not require sample digestion, the digestion criterion was considered not applicable and was excluded from the denominator. Scores were used as descriptive indicators of methodological and analytical quality rather than as formal estimates of risk of bias.

### 2.6. Reference Standards and Assessment of Adequacy of Mineral Levels

Analytically measured mineral concentrations were interpreted in relation to established nutritional guidelines (Tables S3 and S4), including those proposed by the European Pet Food Industry Federation (FEDIAF) [12] and the Association of American Feed Control Officials (AAFCO) [11]. For the comparative assessment performed specifically in this review, FEDIAF 2024 [12] recommendations were selected as the common reference framework because they provide a single, current set of nutritional recommendations for complete dog and cat foods and allow consistent comparison across the dietary categories included in the review. When individual diet concentrations were available, they were therefore classified as below, within, or above the corresponding FEDIAF recommended range. These classifications formed the basis of a heatmap. AAFCO recommendations were retained only when reporting or discussing adequacy assessments made by the original studies; in such cases, the reference framework is explicitly identified in the text and these classifications were not combined with the FEDIAF-based heatmap assessment.

### 2.7. Data Synthesis and Presentation

Analytically derived mineral composition data were summarised in separate tables for dog (Table 2 and Table 3) and cat (Table 4 and Table 5) diets, and for macrominerals and trace elements, organised on the basis of the main dietary factors evaluated in the studies (diet type, dietary format, formulation characteristics, and physiological context). Information on potentially toxic elements was summarised separately due to the limited number of studies available and the high heterogeneity in the elements evaluated, analytical scope, and reporting approaches. Direct quantitative comparisons of absolute mineral concentrations across studies were restricted to data expressed on the same basis.

To provide a relative measure of between-product variability, coefficients of variation (CVs) were calculated as (SD/mean) × 100 for mineral concentrations reported as mean ± SD or for which mean and SD could be directly derived as described above. CVs were calculated within each study or dietary subgroup and were not estimated when SD was unavailable. The resulting CVs are presented in Table 6.

To provide an integrative visual summary of mineral imbalances across diet types and species, a heatmap was constructed using analytically measured mineral composition data reported at the level of individual complete diets. Only analytically based studies providing mineral concentrations for individual diets and allowing comparison with FEDIAF 2024 reference values were included. For each mineral and dietary category, individual values were classified as being below, within or above the recommended range, and the heatmap represents the percentage of diets outside the recommended range. Each row of the heatmap represents one study or one dietary subgroup within a study; therefore, each cell is derived from a single study. The number of individual diets contributing to each row is reported as *n* in the figure. No AAFCO-based classifications were incorporated into the heatmap. Single-diet clinical case reports were excluded from the heatmap.

Because data on potentially toxic elements could not be summarised using the same approach as for essential minerals—owing to differences in study design, analytical coverage, reporting criteria and toxicological reference values—a separate semi-quantitative heatmap was generated. Only studies evaluating commercially available pet foods were included in the synthesis. For each study and element, evidence was classified on the basis of the reported level of regulatory non-compliance or toxicological concern. When available, the proportion of samples exceeding the corresponding reference thresholds was included in the visual classification by using a graded colour scale. Consequently, the heatmap should be interpreted as a comparative representation of reported toxicological concern rather than a direct comparison of regulatory compliance across studies.

For the purposes of the descriptive synthesis, dietary categories were operationally defined according to their formulation and mode of preparation. Conventional commercial diets comprised commercially manufactured complete foods, including dry and wet products; cereal-free or grain-free commercial formulations were considered formulation variants within this category rather than alternative diets. The term “alternative diets” was retained only as a broad organisational label encompassing RMBDs, home-prepared diets, and plant-based diets, which depart from conventional commercial feeding patterns in their formulation and/or preparation. Because these dietary approaches differ substantially in composition and formulation strategy, they were analysed and discussed as separate subtypes and were not quantitatively pooled as a single dietary category. RMBDs were defined as diets based predominantly on uncooked animal-derived ingredients, including meat, edible bone and/or organs, whether commercially manufactured or owner-formulated. Home-prepared diets were defined as non-commercial recipes prepared from individual ingredients; when reported by the original studies, professionally formulated or veterinary-prescribed diets were distinguished from owner-formulated or internet-sourced recipes. Commercial products described as “homemade-style” were identified separately and were not classified as home-prepared diets. Plant-based diets comprised commercial formulations based predominantly or exclusively on plant-derived ingredients; vegetarian or vegan home-prepared recipes were retained within the home-prepared category and identified as such.

## 3. Results and Discussion

**Figure 1:**
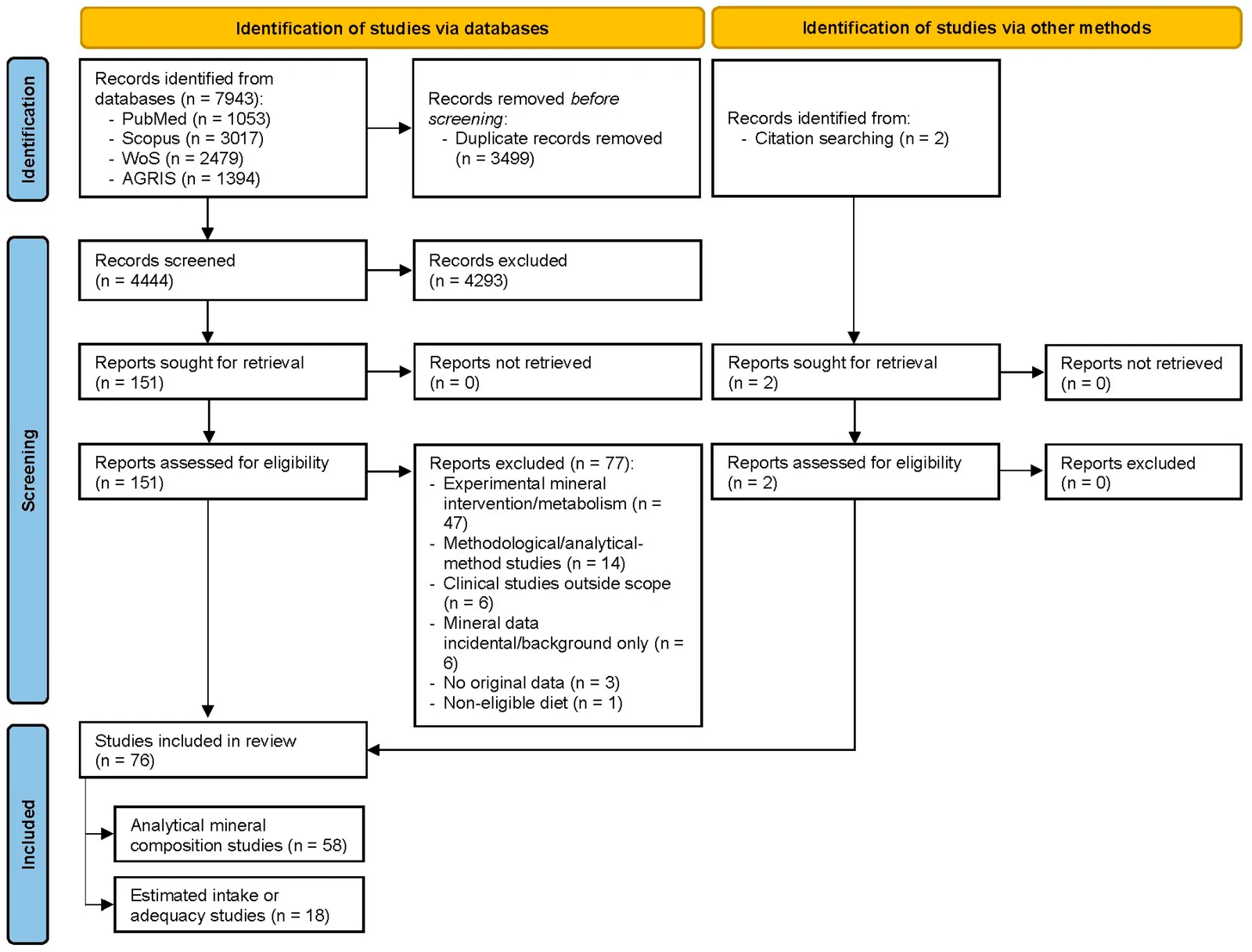
PRISMA [16] flow diagram outlining the multi-database systematic literature search, screening process, and eligibility assessment.

The research strategy identified 7943 records, of which 3499 were duplicates. Following screening and eligibility assessment, 76 studies were included in the review: 58 analytical studies evaluating mineral composition and 18 studies assessing estimated mineral intake or adequacy of mineral levels. The complete study selection process is presented in Figure 1.

**Figure 2:**
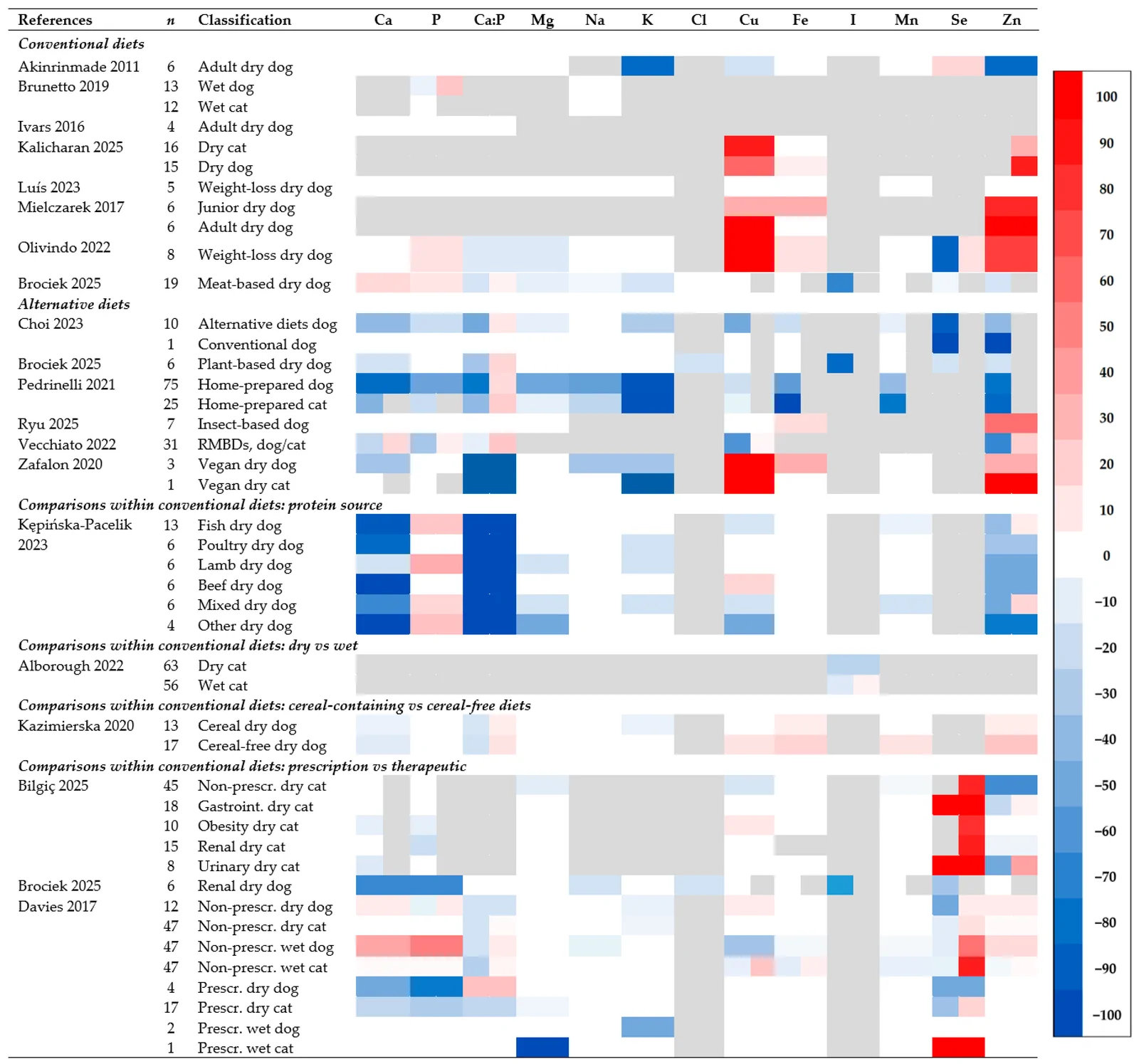
Heatmap representation of mineral imbalances in dog and cat diets relative to FEDIAF 2024 guidelines. Each row represents one study or one dietary subgroup within a study, and the number of individual diets contributing to each row is indicated as *n*. Colour intensity represents the percentage of individual diets falling outside the applicable FEDIAF threshold(s). Blue (−100% to 0%) indicates the percentage of diets below the recommended minimum and red (0% to 100%) indicates the percentage above the applicable maximum, with darker shades representing a higher proportion of diets. White indicates 0% for the corresponding direction of deviation (below or above), whereas grey indicates that the mineral was not assessed, data were unavailable, or the reported basis was not directly comparable with the applicable FEDIAF threshold. When both lower and upper thresholds were assessable, the cell is split, with the left half representing the percentage below the minimum and the right half the percentage above the maximum. References represented in this heatmap, in order of appearance from top to bottom, are [18,19,23,25,26,28,31,43,44,45,48,50,52,54,57,60,71,73]. Gastroint.: gastrointestinal; Prescr.: prescription; RMBDs: raw-meat-based diets.

The analytical mineral composition data extracted from the reviewed studies are summarised in Table 2, Table 3, Table 4 and Table 5. Weight-based values are expressed using harmonised units, predominantly on a dry-matter basis, whereas values expressed per 1000 kcal ME are underlined in the tables. Specifically, data on dog diets are presented in Table 2 (macrominerals) and Table 3 (trace elements), and data on cat diets are presented in Table 4 (macrominerals) and Table 5 (trace elements). To facilitate interpretation of the adequacy of mineral levels across dietary categories, the analytical results were included in the visual synthesis presented in Figure 2.

### 3.1. Overview of Analytical Studies on Mineral Composition of Dog and Cat Diets

This section synthesises evidence from studies that have directly quantified the mineral composition of complete diets formulated for dogs and cats, forming the empirical basis of the present analysis. The available literature comprised studies analysing dog diets exclusively (n = 22), studies including both dogs and cats (n = 26), and a smaller number of studies focused solely on cat diets (n = 10). Overall, dogs were represented in a greater number of studies than cats (48 and 36 studies, respectively), and dog diets were also represented across a broader range of diet types, formats, and formulation strategies.

A broad spectrum of dietary categories was evaluated across the studies, including conventional commercial diets, alternative dietary approaches (namely RMBDs, home-prepared, and plant-based diets), and selected veterinary therapeutic diets. Commercial cereal-free or grain-free formulations were considered variants of conventional commercial diets rather than a separate alternative dietary category. Most studies adopted a descriptive design focused on a single dietary category, while comparatively few directly compared different diet types, dietary formats or formulation approaches within the same experimental framework. A high degree of heterogeneity was also observed in dietary format (dry extruded versus moist canned products), ingredient composition, and life stage or physiological context, reflecting the diversity of feeding practices addressed in the available literature.

Regarding mineral coverage and data structure, calcium and phosphorus were the most frequently reported macrominerals, whereas iron, zinc, copper, iodine, and selenium were the most commonly analysed trace elements. Limited data were available for other trace elements, and the spectrum of minerals analysed varied substantially across studies. Reporting resolution also differed, with some studies providing mineral concentrations at the level of individual diets and others reporting only aggregated values, contributing to additional heterogeneity in the available evidence.

Beyond differences in reporting resolution and mineral coverage, the methodological quality of the studies also varied widely. The methodological and analytical quality assessment (Table S5) showed substantial variation among studies. Overall, analytical methods, sample description, and result reporting were generally well documented, whereas important weaknesses were identified in analytical quality-control procedures and sample representativeness.

The limited use of certified reference materials or recovery studies to evaluate analytical accuracy (24/58 studies) was a particularly important finding. While several studies reported duplicate or triplicate analyses (36/58 studies), which provide information on analytical precision, fewer studies demonstrated analytical accuracy through validation procedures. This distinction is especially important because many studies aimed to determine compliance with nutritional recommendations. The absence of analytical accuracy validation thus reduces confidence in the absolute accuracy of some reported concentrations.

Substantial variation in the procedures used to ensure sample representativeness was also observed. Most studies analysed only a single package of each product, whereas a minority combined multiple packages into composite samples (11/58 studies). Among the latter, some studies sampled packages from the same production batch, whereas others deliberately included different batches. Similarly, homogenisation procedures ranged from detailed protocols involving subsampling, grinding, pooling, and homogenisation (29/58 studies) to brief statements indicating only that samples were ground or milled (14/58 studies). Several studies did not provide any information about homogenisation. Because the representativeness of analytical results depends on both the original sample and the homogeneity of the analytical subsample, insufficient reporting of either sampling or homogenisation procedures reduces confidence that measured mineral concentrations accurately reflect the composition of the final product.

Finally, the limits of detection or quantification were infrequently reported (19/58 studies). This limitation is unlikely to be important for the major minerals typically present at concentrations greatly exceeding analytical detection limits. However, it may be more important for trace minerals and potentially toxic elements, for which the analytical sensitivity directly influences the interpretation of non-detection and compliance assessments.

Altogether, these methodological limitations should be considered when interpreting reported mineral concentrations and evaluating compliance with nutritional recommendations, as some of the variability observed across studies may reflect methodological differences rather than true differences between diets. Nevertheless, analytical studies provide a valuable but heterogeneous, unevenly distributed overview of mineral composition in dog and cat diets. While the available data enable identification of broad patterns and ranges of mineral variability, differences in study design, analytical quality-control procedures, sampling strategies, reporting resolution, and coverage across species and diet types limit direct comparability across dietary categories. These aspects are explored in the following sections, which examine dietary and physiological factors influencing mineral variability and adequacy.

### 3.2. Macrominerals

Regarding macromineral composition, differences in average concentrations between conventional and alternative diets were not consistent across studies and did not follow a uniform directional pattern. Both diet categories encompassed wide ranges of calcium and phosphorus values, with a substantial overlap. There was a similar lack of consistent differences between diet types for other macrominerals, including magnesium, sodium, and potassium, although the patterns were generally less pronounced than for calcium and phosphorus. By contrast, available information on chloride was scarce and insufficient to allow robust comparisons between diet categories.

In this context, between-product variability, expressed as coefficients of variation (CVs), provided an additional measure for comparing mineral composition across dietary categories (Table 6). CVs varied among studies and minerals, although particularly high values for calcium and the calcium-to-phosphorus (Ca:P) ratio were observed in several alternative diet datasets. For example, CVs for calcium exceeded 60% in several RMBD datasets and were even higher in some home-prepared diet groups, reaching 128–134% in dog diets. Similarly, variability in the Ca:P ratio was also particularly pronounced in home-prepared diets, with CVs exceeding 100% in several subgroups. Nevertheless, this pattern was not uniform across all alternative diets, and conventional commercial diets also showed substantial variability for selected minerals. However, high variability was less consistently reproduced across independent conventional-diet studies. Overall, the pronounced variability observed in several alternative diet categories, together with the occurrence of both below- and above-recommended concentrations at the individual product level (Figure 2), indicates a greater potential for mineral imbalance.

The heatmap analysis broadly supported this pattern, although the visual prominence of some deviations in conventional diets should be interpreted cautiously because several rows represent subgroups from the same study. In particular, the marked calcium and Ca:P deficiencies across protein-source categories were largely derived from a single study [48]. Other independent surveys of conventional commercial diets reported no calcium or phosphorus concentrations below recommended minimum values [40] or concentrations within permitted ranges [72], although specific problems, such as high phosphorus concentrations and low Ca:P ratios, were identified in subsets of commercial cat foods [67]. Given the critical role of Ca:P balance in skeletal development and bone metabolism, this variability represents one of the most nutritionally relevant differences between diet categories [72].

Beyond analytical determinations, evaluations based on calculated nutrient intake from owner-formulated recipes further support this interpretation, showing that high variability in macromineral concentrations in alternative diets is not limited to isolated products but represents a recurrent feature across a large number of formulations, particularly for calcium, phosphorus, and the Ca:P ratio. In 95 owner-prepared bone and raw food rations, Dillitzer et al. [77] found that 60% contained at least one relevant nutritional imbalance; 10% supplied less than 25% of the recommended calcium allowance and had markedly low Ca:P ratios. Similarly, ration-based assessment by Hajek et al. [81] identified significantly more nutritional imbalances among dogs receiving RMBDs than among commercially fed controls. Together, these findings indicate that macromineral imbalance in alternative diets is not restricted to isolated analytical surveys but recurs across independent datasets and assessment approaches.

Within the group of alternative diets, differences in macromineral concentrations were observed between specific subtypes. The calcium and phosphorus contents of raw meat-based diets were highly variable, with frequent imbalances in the Ca:P ratio, which may partly reflect the proportion and type of bone included in the formulation (Table 2 and Table 4). Consistent with this pattern, analytical studies reported substantial variability in calcium supply and disturbances of the Ca:P ratio in RMBDs [28,71]. Estimated-intake studies provide complementary evidence, showing that mineral imbalances also occur frequently in owner-formulated raw diets, including diets based on information obtained from non-specialist sources and diets incorporating commercial or natural supplements [77,81]. Importantly, RMBDs were not characterised by a uniform calcium deficiency: both deficient and excessive calcium supply and deviations of the Ca:P ratio in both directions were reported. Thus, the principal concern appears to be the unpredictability of calcium supply and Ca:P balance rather than a single directional deviation.

Home-prepared diets showed one of the clearest patterns of macromineral inadequacy. While recommended levels of macrominerals were almost reached in some formulations, large deviations from these levels were observed in others, resulting in wide dispersion and high coefficients of variation [57]. As observed for RMBDs, large-scale assessments of home-prepared diet recipes based on estimated intakes further indicate that inadequate levels of macrominerals, particularly calcium and phosphorus, are highly prevalent and may be related to imprecise ingredient quantification and inadequate supplementation strategies [89,92].

Plant-based diets require a more nuanced interpretation. Commercial plant-based formulations did not uniformly show poor macromineral adequacy, and their performance differed among studies and products. Nevertheless, deficiencies of calcium and phosphorus and disturbances of the Ca:P ratio were documented in several independent datasets, particularly in products formulated for growth or all life stages [25,33,73]. For example, in Dodd et al. [33], only two of seven plant-based dog foods intended for growth or all life stages met all FEDIAF minimum mineral recommendations, with calcium and phosphorus insufficiencies particularly evident among the wet products. In the Brazilian series evaluated by Zafalon et al. [73], all three vegan dog foods had Ca:P ratios below FEDIAF recommendations, while the single vegan cat food had a low Ca:P ratio; however, the small number of products limits generalisation from that study. These findings indicate that the nutritional adequacy of plant-based diets depends strongly on formulation and supplementation rather than on the absence of animal-derived ingredients per se.

### 3.3. Microminerals

Micromineral composition showed marked variability across studies and dietary categories, with differences between diet types in both mean values and variation (Table 3 and Table 5). The mean concentrations of microminerals in conventional maintenance diets were often within or close to recommended ranges, although substantial variability and both below- and above-recommended concentrations were reported for specific minerals and dietary subcategories. Thus, conventional diets did not show a uniform pattern of either deficiency or excess, and deviations appeared to be largely study-, mineral-, or product-specific (Figure 2).

The micromineral profiles of alternative diets were highly heterogeneous, with wide ranges of mean values across studies and frequent deviations from recommended ranges (Figure 2). However, these deviations did not follow a consistent directional pattern. Deficiencies were prominent in several independent datasets, particularly for copper, iron, manganese, selenium, and zinc, whereas other studies reported excesses or a combination of concentrations below and above recommended levels. As observed for macrominerals, the mean values often masked substantial dispersion at the individual product level [28,57,71,73]. Consequently, differences between conventional and alternative diets were better characterised by the heterogeneity and recurrence of mineral imbalances across formulations than by consistently higher or lower mean micromineral concentrations. Assessment of the variability further emphasized these differences. Coefficients of variation for micromineral contents varied widely in both conventional and alternative diets. Very high CVs were also observed in individual conventional datasets, including Mn in [18], Zn in [44], and Se in [54], indicating that high variability was not exclusive to alternative feeding strategies (Table 6). Nevertheless, several alternative-diet datasets—particularly home-prepared and commercial homemade-style/RMBD formulations—showed high CVs simultaneously across multiple microminerals, suggesting less consistent mineral provision across products within these groups (Table 6). The heatmap analysis similarly showed both deficiencies and excesses in the two broad dietary categories, but deficiencies affecting several microminerals recurred across multiple independent alternative-diet studies (Figure 2). Thus, the principal distinction was not a simple conventional-versus-alternative difference in the direction of mineral concentrations, but rather the recurrence of broader, multielement imbalances in some alternative diet categories.

Distinct micromineral patterns were also evident among alternative diet subtypes. Commercial home-made style/RMBDs formulations showed highly heterogeneous profiles, with both deficient and excessive concentrations reported for several trace elements, particularly copper and zinc, rather than a consistent directional pattern [28,71]. Home-prepared diets showed pronounced variability together with frequent deficiencies affecting multiple microminerals, especially iron, zinc, manganese, and copper [57]. This pattern was also supported by estimated-intake studies, in which multiple mineral inadequacies were common in owner-formulated diets for both dogs and cats [89,92]. Plant-based and vegan diets also showed considerable between-product variability, with adequacy depending strongly on formulation and supplementation rather than following a uniform pattern across products [33,73].

### 3.4. Dietary Format and Processing: Dry Versus Moist Diets

One of the features of conventional commercial diets most frequently examined in the reviewed studies is the influence of physical form, particularly the comparison between dry and wet formulations, which differ in composition and technological processing. This comparison has attracted attention due to concerns regarding differences in mineral concentrations and between-product variability. Several analytical studies included both dry and wet or canned foods within the same study [19,22,31,36,56,61,63,66,67,68]. Within-study comparisons are particularly informative because they reduce, although do not eliminate, heterogeneity related to analytical methods, geographical origin, and study design. Importantly, these studies differed in the method of expressing mineral concentrations, with values reported either on a dry matter or an as-fed basis. This aspect must be considered when interpreting apparent discrepancies between studies, as the moisture content affects both macro- and micromineral concentrations due to a dilution effect.

When macrominerals were considered, some within-study comparisons showed higher concentrations of several elements in wet foods. Davies et al. [31] reported higher dry-matter concentrations of calcium, phosphorus, sodium, and potassium in wet than in dry foods for both dogs and cats. Similarly, Sgorlon et al. [61] found significantly higher calcium, phosphorus, sodium and potassium in moist canned than in dry extruded dog foods. However, this pattern was not consistent across all studies. Paulelli et al. [56] reported higher calcium, phosphorus, and magnesium in dry than in canned foods, although the unclear basis of expression limits direct interpretation of this contrast. Furthermore, in energy-normalised comparisons of commercial cat foods, Summers et al. [67] found no significant effect of food format on phosphorus or the Ca:P ratio, while canned foods contained significantly less magnesium than dry foods. A related study of senior cat foods similarly found no significant dry–canned differences in phosphorus, calcium, or Ca:P ratio, although magnesium was lower in canned foods [66]. Thus, dietary format was associated with differences in macromineral composition, but there was no uniform direction of effect across minerals or studies.

The concentrations of microminerals were also highly element-specific. Paulelli et al. [56] reported significantly higher iron and selenium concentrations in canned foods for both dogs and cats, as well as higher copper and zinc concentrations in canned dog foods. In contrast, Sgorlon et al. [61] found significantly higher copper and zinc concentrations in dry extruded dog foods, whereas iron, manganese, and selenium did not differ significantly between formats. In cats, Summers et al. [68], using an energy-normalised basis, found no significant difference in copper between dry and canned foods, whereas zinc concentrations were significantly higher in dry foods; iron did not differ significantly between the two formats. Iodine showed a different pattern: Alborough et al. [19] found higher mean iodine concentrations and a wider range in wet than in dry cat foods. For selenium, Simcock et al. [63] found that wet cat foods had both higher and substantially more variable concentrations than dry cat foods and dog foods, whereas wet and dry dog foods showed similar selenium concentrations. The authors related the higher selenium concentrations in wet cat foods to the greater representation of seafood-based products, illustrating that an apparent association with dietary format may partly reflect ingredient composition. Overall, the available evidence does not support a general distinction in which wet foods are consistently richer or poorer in trace minerals than dry foods; rather, the direction and magnitude of the differences depend on the mineral, species, and formulation.

Beyond differences in mean concentrations, variability also differed according to mineral and study, without a consistent pattern indicating greater dispersion in one feeding format (Table 6). Although wet foods showed higher coefficients of variation for several minerals in some datasets, the opposite occurred for others. For example, in Sgorlon et al. [61], wet dog foods showed higher CVs for calcium, phosphorus, potassium, copper, iron, manganese, and selenium, whereas dry foods were more variable for magnesium, sodium, cobalt, and zinc, and variability in the Ca:P ratio was similar between formats. Paulelli et al. [56] also showed marked differences in variability between dry and canned foods, but their direction depended on the mineral and species, while Duran et al. [36] reported substantially lower variability for iron in wet than in dry foods. Other studies likewise reported broad ranges of mineral concentrations in both formats [36,63]. Overall, substantial between-product variability occurs in both dry and wet foods, and the available evidence does not support interpreting either format as systematically more standardised than the other. The available evidence further illustrates the element-specific nature of deviations from mineral recommendations in dry and wet commercial diets. Within the studies represented, dry and wet foods showed different patterns of deviation from FEDIAF recommendations rather than a uniform advantage of one format over the other. For macrominerals, deviations in calcium and phosphorus occurred in both formats, with some wet-diet groups showing more frequent values above recommended levels, whereas below-range values were also observed in dry foods. Deviations in the Ca:P ratio similarly varied among species and dietary groups. Among microminerals, selenium showed some of the most marked format-related differences, with excess values occurring particularly in wet cat foods, while below-recommended concentrations were also observed in dry foods. In the heatmap, iodine showed a different pattern, with below-minimum concentrations occurring more frequently in dry cat foods in Alborough et al. [19], whereas wet foods included both below- and above-range values. Copper, iron, and zinc also showed heterogeneous patterns depending on species and study.

Overall, the available data indicate that dry and wet commercial diets can differ substantially in mineral composition, but dietary format alone is not a reliable predictor of mineral concentration, variability, or nutritional adequacy. The observed differences are likely to reflect the combined influence of ingredient composition, formulation and supplementation practices, and the technological requirements associated with each format. Because the available studies did not experimentally separate these factors, specific effects of extrusion versus canning cannot be distinguished from the compositional differences between the products subjected to these processes.

### 3.5. Ingredient Composition, Formulation Quality and Market Positioning

Beyond diet category and format, ingredient composition and market positioning were also associated with differences in mineral composition. Several comparative studies addressed these factors, but their designs and classification criteria were heterogeneous, with some interpretations necessarily based on a limited number of studies, and the conclusions must therefore be interpreted with caution.

Kępińska-Pacelik et al. [48] specifically evaluated the influence of the main animal-derived ingredient on mineral composition in conventional dry dog foods, classified according to fish, poultry, lamb, beef, mixed, and other sources. No clear clustering of diets according to ingredient type was observed when the overall mineral profile was considered. Indeed, principal component analysis indicated that the main animal source did not clearly explain the overall mineral profile and did not significantly affect mineral ratios, despite significant differences between ingredient groups for individual elements [48]. The absence of a clear ingredient-defined pattern is also evident in the heatmap (Figure 2), although the different ingredient categories represented there derive from the same study and should therefore not be regarded as independent evidence. Within this dataset, calcium concentrations were frequently below recommended levels across ingredient groups, whereas phosphorus was generally adequate or elevated, resulting in frequent disturbances of the Ca:P ratio. Thus, the main animal ingredient did not provide a reliable predictor of overall mineral adequacy.

Brociek et al. [25] found broadly comparable mineral profiles between plant- and meat-based dog foods for most elements, although plant-based products contained more potassium and less iodine. Flavour- or ingredient-associated effects were also reported in other datasets, but again differed by element. Altınok-Yipel et al. [20] identified differences among wet cat foods labelled as salmon, tuna, liver, or other aquatic products, although no consistent pattern extended across all elements and interpretation was complicated by differences in life-stage designation. Seafood-flavoured foods had the highest selenium concentrations in Simcock et al. [63], whereas the presence of fish ingredients was not significantly associated with iodine concentration in Edinboro et al. [37], and Alborough et al. [19] similarly found no significant association between fish flavour and iodine concentration. Overall, these findings indicate that ingredient composition can influence individual minerals, but broad ingredient or flavour categories do not reliably predict the overall mineral profile or nutritional adequacy. Market price category has also been explored as a potential proxy for formulation strategy. From a consumer perspective, lower-priced products are often perceived as being of inferior quality, whereas premium and super-premium diets are commonly associated with superior nutritional value. However, evidence from three independent studies does not support a simple relationship between price or commercial tier and mineral adequacy [43,51,58]. Ivars et al. [43] compared super-premium, premium, and white-brand dog foods and found that all products met the minimum requirements for the nutrients assessed on an energy basis, despite a 66.9% difference in estimated daily feeding cost between the most expensive and cheapest products. Notably, one white-brand food had the highest calcium and phosphorus concentrations. Macías-Montes et al. [51] similarly found no consistent advantage of premium over low-cost foods: in dogs, iron and copper were higher in low-cost products whereas zinc was higher in premium products, with no significant differences for selenium or manganese; in cats, only iron differed significantly and was higher in low-cost products. Pereira et al. [58], using low- (≤€1.20/kg), medium- (>€1.20 to ≤€4.07/kg), premium- (>€4.07 to ≤€5.30/kg), and super-premium (>€5.30/kg) categories, likewise observed differences in mineral concentrations without a monotonic improvement with increasing price; low-priced diets had the highest mean calcium and phosphorus concentrations, whereas super-premium diets had the lowest mean values for both minerals. Commercial positioning may therefore be associated with different formulation strategies, but higher price does not ensure closer alignment with mineral recommendations. Taken together, these studies indicate that price or commercial tier is a poor proxy for mineral composition or nutritional adequacy and that the observed differences more likely reflect product-specific formulation and supplementation practices [43,51,58].

Formulation practices and supplementation were also important determinants of the final mineral profile. In Kazimierska et al. [45], declared trace-element additives accounted for approximately 3–86% of the total analytically measured concentrations, indicating that both intrinsic mineral content of raw materials and added mineral sources contributed substantially to the final profile. This distinction was particularly evident in home-prepared diets. Pedrinelli et al. [57] found lower calcium concentrations and Ca:P ratios in non-supplemented dog and cat recipes, together with lower sodium and iron in non-supplemented feline diets; however, the presence of a supplement alone did not ensure nutritional adequacy, and increasing the number of ingredients likewise did not guarantee a balanced formulation. Estimated-intake evidence supports the same interpretation: feline recipes written by veterinarians, as well as recipes including vitamin–mineral supplements, contained fewer nutrients below NRC recommended allowances than recipes from non-veterinary sources or those without supplements; nevertheless, none of the evaluated recipes met all recommended allowances [92]. Together, these findings indicate that formulation quality depends not simply on the presence of particular ingredients or supplements, but on their quantitative integration within the complete diet.

However, interpretation of apparent mineral oversupply based on total concentrations alone should also consider bioaccessibility and bioavailability. The fraction of a mineral released from the food matrix and subsequently absorbed may vary according to its chemical form, ingredient matrix, interactions with other dietary components, and source of supplementation [93,94,95]. Consequently, concentrations exceeding recommended levels do not necessarily translate into a proportional increase in biologically available mineral [93,94]. This distinction has been demonstrated directly in pet foods: simulated gastric extraction released substantially lower proportions of several minerals than total acid digestion [93], while selenium accessibility differed according to diet composition and processing [94]. In vivo evidence further indicates that mineral utilization cannot always be predicted from dietary intake alone, as demonstrated for selenium in canned versus kibble diets [96] and for organic versus inorganic zinc sources in dogs [95]. This does not eliminate the potential relevance of chronic oversupply, particularly for elements with established maximum levels or a propensity for tissue accumulation, but highlights the limitations of assessing nutritional adequacy solely from total mineral concentrations. Most studies included in the present review quantified total mineral concentrations rather than directly assessing bioaccessibility or absorption; therefore, the extent to which differences in total concentrations translated into differences in biologically available mineral could generally not be determined.

Altogether, these studies highlight that ingredient choice, formulation control, and market positioning all contribute to the variability in mineral composition, but none provides a reliable standalone proxy for overall mineral adequacy. Ingredient-related effects were predominantly element-specific rather than reflecting a consistent advantage of any particular ingredient category. Likewise, premium positioning or higher price did not consistently correspond to a more favourable mineral profile. Consistent mineral supply therefore appears to depend on the combined effects of raw-material selection, quantitative formulation, appropriate supplementation, and quality assurance throughout the production process.

### 3.6. Physiological Context and Feeding Level

Physiological status and feeding level represent critical but often underappreciated determinants of mineral adequacy in companion animal diets. Mineral requirements vary substantially across life stages and physiological states, including growth, gestation, lactation, and adult maintenance. They may also be further modified under conditions of energy restriction or disease. Dietary strategies designed to support specific physiological functions or clinical outcomes can therefore alter both mineral intake and long-term exposure. Despite the broad range of physiological contexts that may influence mineral requirements, available analytical evidence in companion animals remains limited, with studies focusing primarily on growth, ageing, energy-restricted feeding, and selected pathological conditions.

Analytical evidence specifically addressing mineral adequacy across physiological life stages is relatively limited but is not restricted to growth. Alvarado et al. [21] evaluated 26 commercial dry diets formulated for growing dogs and found that 96.2% met recommended concentrations for calcium, phosphorus, and magnesium, whereas adequacy was less frequent for sodium and zinc (80.8%) and particularly potassium (34.6%). Overall, only 23% of the diets fulfilled all evaluated AAFCO nutrient specifications. Precise formulation is important as skeletal development during growth is highly sensitive to the Ca:P ratio. Additional evidence from Mielczarek and Szydłowski [52] showed different mean concentrations of copper, iron, and zinc between junior and adult dog foods, further illustrating that products marketed for different life stages may differ in mineral profile.

Life-stage classification itself also influences the interpretation of adequacy. In the FEDIAF-based assessment of the insect-based products reported by Ryu et al. [60], foods formulated for adult maintenance showed relatively few macromineral deviations, whereas products labelled for all life stages showed substantially more deviations when assessed against the more demanding growth/reproduction profile, particularly for calcium, phosphorus, and the Ca:P ratio. This illustrates the importance of applying a reference profile that corresponds to the intended life stage rather than interpreting a product against adult-maintenance criteria alone.

Evidence is also available for diets marketed for older animals. Summers et al. [66] found no significant differences in the concentrations of phosphorus, calcium, magnesium, sodium, or potassium between foods marketed for senior and adult cats; all senior foods met AAFCO adult-maintenance minimum values for the minerals evaluated. However, substantial within-category variability remained. Similarly, manufacturer-reported data for 37 commercial senior dog foods showed a wide sodium range and an approximately three-fold range in phosphorus concentrations [82]. Thus, a “senior” designation does not appear to correspond to a standardized mineral profile. Dietary management of obesity and weight loss may introduce a distinct risk of mineral inadequacy, particularly because reduced feeding levels can decrease absolute mineral intake even when diet composition appears adequate. Studies evaluating commercial weight-loss diets for dogs have demonstrated that, when feed rations are restricted to achieve a caloric deficit, the absolute intake of selected minerals may fall below recommended levels [50,54]. This distinction was particularly evident in Luís et al. [50], whose diets were generally adequate when evaluated on a compositional basis, whereas estimated intake during energy restriction identified shortfalls particularly for magnesium and potassium. Olivindo et al. [54] similarly found that seven of eight veterinary weight-loss diets supplied at least one nutrient below NRC and FEDIAF recommendations under several modelled restriction scenarios, with calcium, magnesium, potassium, selenium, and sodium among the minerals affected.

Importantly, this apparent discrepancy highlights a fundamental limitation of concentration-based nutritional assessment. Evaluations based on heatmap analysis capture dietary formulation and relative mineral density, whereas intake-based studies account for real feeding levels and physiological context. The same pattern is supported by several intake-based studies [79,80,84,86]. In the theoretical analysis by Linder et al. [86], none of the five diets was inadequate when expressed per 1000 kcal, yet every diet supplied at least one essential nutrient below the NRC recommended allowance when energy intake was reduced to 79 kcal/kg BW^0.75/day, with selenium among the nutrients most frequently affected. German et al. [79] likewise found that selenium, magnesium, and potassium intakes could fall below NRC recommended allowances during long-term canine weight loss, although—with few exceptions—essential nutrients remained above minimum requirements and no clinical signs of mineral deficiency were observed. With more moderate restriction, Keller et al. [84] identified selenium and potassium intakes below NRC recommendations only in a minority of dogs and cats. Cats show a similar distinction between recommended intake and overt deficiency. In German et al. [80], selenium intake was below NRC recommendations in a substantial proportion of cats and below FEDIAF recommendations in 72%, whereas potassium was below recommendations in only a small proportion. Taken together, these studies support describing such findings as potential intake inadequacy rather than established mineral deficiency. The likelihood of an inadequate intake depends on the degree of energy restriction, the nutrient density of the diet, the species, and the duration for which restricted feeding is maintained. Whether similar concerns apply to long-term maintenance at chronically low energy allowances remains insufficiently studied. The strongly context-dependent nature of mineral adequacy in companion animal diets is further illustrated by pathological conditions and therapeutic feeding. In these contexts, diets are intentionally formulated to modify mineral supply in accordance with specific clinical objectives and therefore purposely differ from standard maintenance recommendations. Analytical studies have evaluated renal, hepatic, gastrointestinal, hypoallergenic, urinary, obesity-management, and enteral diets in dogs and cats [23,25,40,55,59], although study designs and clinical indications differ considerably.

Diets formulated for renal support provide one of the clearest examples of purposeful modification of mineral composition. Brociek et al. [25] found lower calcium, phosphorus, magnesium, iron, and selenium concentrations in renal-support dog foods than in the other dietary groups; importantly, calcium and phosphorus were excluded by the authors from their overall compliance assessment because their reduction was intentional. Gagné et al. [40] similarly found Ca:P ratios above maintenance recommendations in therapeutic renal foods, while Or et al. [55] reported significantly lower calcium and phosphorus concentrations across prescription dog-food groups than in non-prescription controls. In cats, Bilgiç et al. [23] also found significantly lower phosphorus in renal diets than in non-prescription foods, with 20% of renal products falling below the general FEDIAF minimum used for comparison. The authors explicitly noted that low phosphorus was expected in renal formulations.

Accordingly, deviations shown for prescription diets in Figure 2 should be interpreted as departures from general nutritional reference ranges rather than automatically as evidence of inappropriate therapeutic formulation. When measured against general nutritional guidelines, such diets may appear imbalanced; however, some deviations may reflect purposeful formulation strategies aligned with disease-specific nutritional goals and should not automatically be interpreted as formulation errors. This highlights the limitations of applying uniform adequacy criteria across fundamentally different feeding contexts and underscores the need for condition-specific reference frameworks.

Importantly, chronic kidney disease is not managed exclusively through commercial prescription diets. Home-prepared diets are also used for dogs and cats with chronic kidney disease, including for individualized nutritional management [85]. However, Larsen et al. [85] found that none of the 67 evaluated recipes met all NRC recommended allowances. Selenium, zinc, and calcium were among the minerals most frequently below recommendations, with substantial differences among recipes. Although these findings are derived from estimated nutrient intakes rather than direct chemical analyses, they provide valuable insight into real-world feeding practices and reinforce the need for careful formulation and professional monitoring of the use of therapeutic home-prepared diets.

Beyond renal disease, other pathological conditions further highlight the clinical relevance of long-term mineral exposure. Copper restriction provides a clear example of intentional therapeutic modification. Gagné et al. [40] found copper concentrations below maintenance recommendations in two diets formulated for dogs with hepatic disease, consistent with their intended use in copper-storage disorders. Similarly, Poblanno Silva et al. [90] described successful formulation of a complete home-prepared diet with moderately low copper for a dog with suspected copper-associated hepatopathy, while also illustrating that reducing one mineral without appropriate overall formulation may create other nutrient deficiencies.

Condition-specific differences extend beyond renal and hepatic diets. Or et al. [55] identified distinct mineral profiles among hepatic, renal, gastrointestinal, and hypoallergenic dog foods, with differences involving copper, iron, manganese, calcium, and phosphorus. In cats, Bilgiç et al. [23] likewise found different patterns among gastrointestinal, renal, urinary, and obesity-management diets, including substantial between-group variation in calcium, phosphorus, zinc, and selenium. These findings demonstrate that prescription diets cannot be treated as a nutritionally homogeneous category and that deviations from general maintenance recommendations must be interpreted in relation to their clinical indication.

The available evidence indicates that assessment of mineral adequacy in therapeutic diets requires a framework that explicitly accounts for clinical indication, intended duration of use and feeding level. Evaluating prescription or therapeutic diets outside their disease-specific context may lead to misleading conclusions regarding nutritional adequacy, while prolonged or inappropriate application—whether of commercial or home-prepared formulations—may increase the risk of secondary mineral imbalances. The potential consequences of using a therapeutic diet outside its intended physiological context are illustrated by McMillan et al. [87], in which feeding a renal diet to a growing puppy was associated with low phosphorus supply, an increased Ca:P ratio, reduced bone mineralization, and clinical rickets. This reinforces the need for integrated assessment approaches that consider physiological status, health condition and real-world feeding practices when evaluating mineral balance and long-term exposure in dogs and cats.

### 3.7. Potentially Toxic Elements

**Figure 3:**
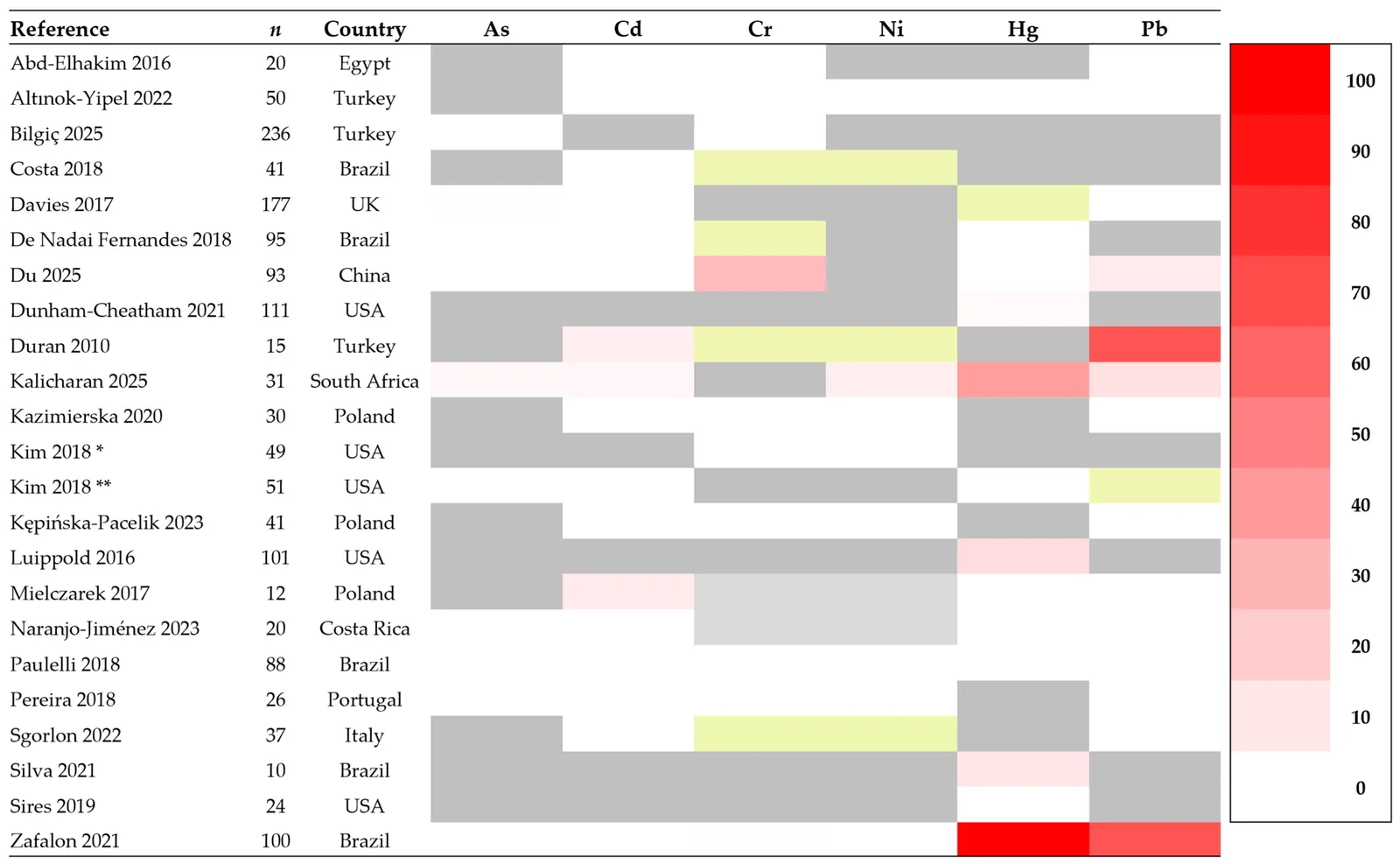
Heatmap summarizing the available quantitative evidence on potentially toxic elements (As, Cd, Cr, Ni, Hg, and Pb) in commercially available pet foods. The number of individual diets contributing to each row is indicated as *n*. White indicates 0% of products above the applicable study-specific regulatory, guideline, or toxicological reference threshold; red shading indicates a non-zero exact percentage, with colour intensity proportional to the percentage (0–100%). Yellow indicates that the element was assessed but an exact eligible product-level exceedance percentage could not be determined, and grey indicates that the element was not assessed. Studies reporting only qualitative toxicological concern without an exact eligible product-level percentage were retained in the narrative synthesis but omitted from the heatmap. Because thresholds and toxicological frameworks differed among studies, the figure should be interpreted as a comparative representation of reported exceedance within each study framework, not as a harmonised comparison of regulatory compliance across studies. The studies included in this figure, in the order shown, correspond to references [17,20,24,30,31,32,34,35,36,44,45,46,47,48,49,52,53,56,58,61,62,64,74]. Kim 2018 * corresponds to reference [46], and Kim 2018 ** corresponds to reference [47].

Compared with the extensive literature addressing essential minerals, evidence regarding potentially toxic elements in pet foods remains relatively limited and heterogeneous in design and interpretation. Twenty-seven analytical studies provided relevant information on potentially toxic elements, of which 23 could be represented in the semi-quantitative heatmap (Figure 3). The studies covered a wide geographical range and varied markedly in sample size, food type, analytical scope, and, importantly, in the toxicological or regulatory frameworks used to interpret the measured concentrations. The reference frameworks were not uniform across studies. Depending on the investigation, concentrations were evaluated against European feed legislation, national pet-food standards, FDA- or NRC-derived maximum tolerable levels, human-derived toxicological reference values, or study-specific exposure metrics such as target hazard quotients or acute hazard indices. For chromium and nickel in particular, several studies measured concentrations without having a directly applicable regulatory maximum for complete pet foods. Consequently, the classifications represented in Figure 3 should be interpreted relative to the reference framework applied in each individual study rather than as a harmonised measure of regulatory non-compliance across all studies. Among the elements investigated, analytical coverage was uneven, with cadmium, chromium, lead, and mercury represented in a relatively large number of studies and arsenic, nickel, or individual element combinations assessed less consistently. Because analytical details differed greatly among studies, the occurrence and toxicological relevance of the evaluated elements were assessed using a semi-quantitative heatmap (Figure 3). This approach identifies reported exceedances or toxicological concern and, when available, the proportion of products exceeding the applicable study-specific reference threshold, thereby providing an overview of reported exceedance patterns across the available literature.

Overall, the available evidence suggests that concentrations of potentially toxic elements in commercial pet foods were frequently below the toxicological or regulatory references applied by the individual studies, but this pattern was not universal. Most study–element combinations with interpretable benchmarks showed either no exceedances or relatively low proportions of affected products, whereas a smaller number of studies accounted for the more conspicuous signals in Figure 3. Mercury and lead produced some of the strongest study-specific concerns. For example, Kalicharan et al. [44] reported exceedances of the applied maximum tolerable levels in 38.7% of foods for mercury and 12.9% for lead, while Silva et al. [62] found one of ten wet cat foods above the European mercury limit. Luippold et al. [49] reported that 13.9% of foods exceeded the comparative 100-ng/g mercury advisory benchmark used in that study, although this was not a pet-food regulatory limit.

The most extreme pattern was reported by Zafalon et al. [74], in which all 100 commercial foods exceeded the FDA-derived maximum tolerable level used for mercury and 67% exceeded the reference applied for lead. This result should not, however, be extrapolated to commercial pet foods as a whole, because similarly high exceedance rates were not consistently reproduced in other datasets and the toxicological benchmarks differed among studies. Zafalon et al. reported mean Hg concentrations well above the MTL they applied and found substantially different patterns between individual toxic elements. Conversely, several surveys reported no relevant benchmark exceedances or toxicological concern for the elements assessed, including studies from Turkey, Poland, Costa Rica, Portugal, and the USA [20,45,53,58,64]. Thus, Figure 3 indicates marked between-study heterogeneity rather than a uniform contamination problem.

Chromium and nickel also require a cautious interpretation. Both were undetectable or below levels of concern in several studies [45,46,48,58], but this pattern was not universal. Du et al. [34] reported chromium concentrations above the Chinese pet-food standard in 25 of 93 products (26.9%), while Kalicharan et al. [44] reported nickel above the study-specific maximum tolerable level in 2 of 31 products.

The association between elevated concentrations of potentially toxic elements and the inclusion of specific animal-derived ingredients is a recurring observation across several studies. Diets containing fish or fish-derived ingredients were more frequently associated with increased concentrations of arsenic and mercury, and in some datasets cadmium, particularly in the study by Kim et al. [47], where fish-based dog foods contained significantly higher As, Cd, and Hg concentrations than poultry- or red-meat-based foods. Luippold et al. [49] also found that all foods exceeding 100 ng/g Hg contained fish as a primary ingredient, with the highest concentrations occurring primarily in wet cat foods. Macías-Montes et al. [51], although not represented quantitatively in Figure 3 because their risk assessment was based on group-level exposure metrics, similarly found significantly higher arsenic and mercury concentrations in fish-based foods.

However, fish ingredients were not a universal predictor of mercury exposure. Sires et al. [64] detected total mercury in only three of 24 canine diets, two of which contained no declared fish ingredients, and concluded that common sources of mercury could not reliably be attributed to seafood. Similarly, Dunham-Cheatham et al. [35] found no single ingredient that consistently explained mercury concentrations when label information and DNA metabarcoding were considered together, although fish DNA was identified in some products with unexpectedly high mercury concentrations. Ingredient source therefore appears to be associated with contaminant exposure, particularly for arsenic and mercury, but labelled flavour or protein source alone is not sufficient to predict the toxic-element profile of an individual food.

Other raw materials may also contribute. Zafalon et al. [74], who analysed ingredients separately from the complete foods, found that animal by-products generally contained higher concentrations of potentially toxic elements than plant-based protein ingredients, while substantial differences were also observed among carbohydrate and fat sources. These findings support an important role for raw-material selection and provenance rather than a simple distinction between animal- and plant-derived ingredients.

No consistent directional effect of food format was evident across the full evidence base. Some mercury-focused studies found that several of the highest or benchmark-exceeding concentrations occurred in cat foods, particularly wet cat foods [35,49], whereas other large studies reported the opposite pattern for several elements. Du et al. [34] found higher mean Pb, Cd, Cr, and As concentrations in dry than in canned foods, with Pb and Cr exceedances confined to dry products, while Zafalon et al. [74] reported higher concentrations of most potentially toxic elements in dry foods. Haydous et al. [42] likewise found higher concentrations of several elements, including Pb, in dry foods, although Cd was higher in wet foods. These contrasting findings indicate that food format itself is unlikely to be a sufficient explanation for contaminant concentrations. Differences in raw materials, geographical origin, supplementation, processing, and other formulation characteristics may all contribute, and their effects cannot generally be separated with the available observational data.

Accordingly, the current evidence does not support describing benchmark exceedances or toxicologically concerning concentrations of potentially toxic elements as a widespread feature of commercial pet foods or an issue confined to a particular diet format. Rather, most datasets showed low or limited concern, alongside a smaller number of studies or products with marked benchmark exceedances. The heterogeneity in analytical methods and toxicological reference values also means that apparently large differences between studies should be interpreted cautiously. Continued surveillance therefore remains warranted, particularly for mercury, lead, arsenic, and chromium, and would benefit from greater harmonisation of pet-specific toxicological reference values and reporting approaches.

The toxicological relevance of total element concentrations may also depend on chemical speciation and species-specific mineral handling. Alborough et al. [97] reported higher renal and urinary arsenic concentrations in domestic cats than in dogs and wildcats; however, renal arsenic concentrations were not associated with chronic interstitial nephritis. Notably, arsenobetaine, an organic arsenic species of relatively low toxicity, was the predominant arsenic form identified in the pet foods evaluated. A similar issue applies to mercury speciation. Dunham-Cheatham et al. [35] found that, although three commercial foods exceeded the comparative reference used for total mercury, estimated methylmercury exposure remained below the NRC chronic exposure level applied by the authors. Sires et al. [64] likewise found methylmercury in only one of 24 canine diets and estimated that the measured concentrations were unlikely to represent a chronic toxicological risk in healthy adult dogs. Thus, total concentrations provide an important screening measure but do not necessarily define the biological or toxicological significance of exposure without information on chemical form, intake, and species-specific handling. These findings further illustrate the limitations of interpreting potential toxicological risk solely from total elemental concentrations.

### 3.8. Limitations and Future Directions

This review also has several limitations that should be acknowledged. The available evidence was highly heterogeneous with respect to study design, dietary categories, minerals evaluated, units of expression, analytical methods and reporting approaches, limiting direct quantitative comparisons across studies and preventing a formal meta-analytic synthesis. Furthermore, many studies reported only summary statistics rather than individual diet values, which restricted some aspects of the comparative analysis and limited inclusion in components of the synthesis that required diet-level data, such as the heatmap assessment of mineral adequacy. The heatmaps should therefore be interpreted as descriptive visual syntheses rather than pooled estimates of the prevalence of mineral inadequacy or toxic-element benchmark exceedance. In Figure 2, each row represents a study or a subgroup within a study; consequently, several visually prominent rows may originate from the same dataset and should not be interpreted as independent replication of a finding. Differences in sample size among rows were also not used to weight their visual contribution. Similarly, the CV analysis in Table 6 was restricted to studies reporting mean and SD, or for which these statistics could be directly derived, and therefore does not provide a complete assessment of between-product variability across the entire evidence base.

Beyond these review-level limitations, important limitations were also identified in the available evidence. Despite the increasing number of studies addressing mineral composition in companion animal diets, important limitations continue to constrain the interpretation and generalization of the available evidence. Analytical data remain unevenly distributed across species, diet types, and physiological contexts. While commercial maintenance diets for dogs and cats are comparatively well represented, analytical information for alternative feeding practices, therapeutic formulations, and specific life stages, particularly gestation, lactation, and ageing, remains limited. This imbalance restricts robust cross-diet comparisons and hampers evaluation of long-term mineral adequacy in less standardized feeding scenarios.

The high degree of methodological heterogeneity among studies poses a major challenge for synthesizing existing evidence. Although numerical units were harmonised across studies, differences in the underlying basis of expression (dry matter, as-fed or energy-normalised) remained when exact conversion was not possible without additional assumptions, limiting direct quantitative comparison across all studies. Furthermore, the representativeness of analysed samples varied widely among studies, with differences in the number of packages evaluated, the inclusion of single or multiple production batches, and the level of detail provided regarding sample preparation and homogenisation. Together, these factors may contribute to part of the variability observed across the literature and affect confidence in reported mineral concentrations. Moreover, mineral concentrations are frequently reported without adequate consideration of feeding level or dietary purpose, increasing the risk of misinterpretation when diets are assessed outside their intended physiological or clinical context.

In addition, dietary composition is rarely considered along with actual mineral intake, particularly in contexts other than standard maintenance conditions. Most analytical studies assess mineral composition independently of actual food intake. By contrast, intake-based investigations demonstrate that reduced food allowance, such as during weight-loss programmes, can result in absolute mineral intakes below recommended allowances even when the composition of the food itself appears adequate. Therapeutic diets pose a different interpretative challenge because their mineral profiles may be intentionally modified for a specific clinical purpose and may therefore deviate from general maintenance recommendations without representing inappropriate formulation. Intake-based evidence remains less common than compositional analysis, and long-term studies linking altered feeding levels or therapeutic formulation with mineral status and clinical outcomes are particularly limited. This highlights the need for integrated approaches that jointly consider formulation strategy, feeding level and long-term exposure.

Potentially toxic elements present a different limitation. Although a substantial number of analytical studies have measured arsenic, cadmium, chromium, nickel, mercury, lead, and related elements, the evidence remains difficult to integrate because studies used markedly different regulatory limits, maximum tolerable levels, toxicological reference values, and exposure-based risk metrics (Figure 3). For chromium and nickel in particular, directly applicable pet-food regulatory limits were not available in several of the frameworks used. Moreover, detection of an element, exceedance of a study-specific benchmark, and demonstration of an actual toxicological risk are not equivalent outcomes. Consequently, the available evidence does not yet allow a harmonised quantitative assessment of chronic exposure risk across commercial pet foods. Establishing species-specific toxicological thresholds through controlled dose–response studies in companion animals may be ethically and practically constrained. Future evidence may therefore need to rely more heavily on observational studies, biomonitoring, clinical data, toxicokinetic information, chemical speciation, and cautious extrapolation from other species.

Beyond total mineral concentrations, current evidence largely overlooks mineral bioaccessibility and bioavailability. Apparently similar total concentrations do not necessarily imply equivalent mineral utilization, and concentrations exceeding recommended levels do not necessarily produce a proportional increase in biologically available mineral. Conversely, this uncertainty should not be interpreted as evidence that chronic oversupply is harmless, particularly for minerals with established maximum levels. Overall, the limited information on mineral speciation, bioaccessibility, and bioavailability remains an important source of uncertainty when nutritional adequacy is inferred solely from total mineral concentrations.

Finally, greater attention should be directed toward trace elements, such as zinc and selenium, with well-established roles in immune regulation, oxidative balance, and cellular homeostasis. While commercial diets are generally formulated to meet current nutritional recommendations, information on whether sustained variability or marginally inadequate levels of trace elements may influence the progression or management of chronic conditions characterized by low-grade inflammation is limited. Addressing this question will require integrated approaches combining dietary assessment, biomarkers of mineral status, and clinically relevant outcomes, rather than isolated evaluation of dietary concentrations.

Future research would benefit from a more holistic framework integrating analytical composition, feeding level, physiological status, bioavailability, and long-term exposure. Harmonization of analytical methods, validation procedures, sampling strategies, and reporting standards, together with increased focus on underrepresented diets, life stages, and health conditions, will be essential to advance the understanding of mineral adequacy and safety in companion animal nutrition.

## 4. Conclusions

The findings of this systematic review highlight that mineral composition and adequacy in dog and cat diets vary in relation to diet type, formulation strategy, processing format, and feeding context, whereas the influence of processing format appears to be more element- and study-specific. Differences in average mineral concentrations between conventional and alternative diets did not follow a uniform directional pattern, and substantial variability was observed in both categories. Nevertheless, recurrent multielement imbalances were documented across several independent alternative-diet datasets, especially in home-prepared diets and some RMBD and plant-based formulations, while particularly high variability in calcium, phosphorus, and the Ca:P ratio was observed in several home-prepared and RMBD datasets. In conventional commercial diets, mineral deviations were also observed, but tended to be more study-, product-, or element-specific. Market positioning or price did not consistently predict improved mineral balance. Ingredient composition influenced particular minerals, but broad ingredient or flavour categories likewise did not reliably predict overall mineral adequacy.

Potentially toxic elements showed a similarly heterogeneous pattern. Most study–element combinations with interpretable benchmarks indicated limited or no toxicological concern, whereas a smaller number of datasets showed substantial exceedances, particularly for mercury and lead. Ingredient composition, especially the use of fish-derived raw materials for some elements, was associated with contaminant concentrations in several studies, but neither ingredient category nor dry versus wet format consistently predicted toxic-element exposure. Importantly, these findings demonstrate that mineral adequacy cannot be reliably assessed solely on the basis of compositional data. Intake-based evidence shows that feeding level and physiological or clinical context can substantially modify nutritional adequacy. Energy restriction during weight-loss programmes may reduce absolute mineral intake despite apparently adequate nutrient concentrations, whereas therapeutic diets may intentionally deviate from general maintenance recommendations to achieve disease-specific nutritional objectives. Altogether, this evidence supports the need for integrated assessment frameworks that account for both dietary concentrations and practical feeding regimes. Interpretation of the available literature should also consider the methodological heterogeneity among studies, particularly regarding analytical validation and sample representativeness, basis of expression, and availability of individual diet-level data.

Future research should prioritize harmonized analytical approaches, improved reporting standards, and expanded coverage of underrepresented diets, life stages, and pathological conditions, in order to support evidence-based nutritional guidance and enhance long-term health outcomes in companion animals.

## Figures and Tables

**Table 1:** Methodological and analytical quality assessment tool for studies evaluating mineral concentrations in dog and cat diets. Each item was scored as 1 when the criterion was met and 0 when absent or insufficiently reported. AF: as fed; CRM: certified reference material; DM: dry matter.

Domain	Item
Sample representativeness	c1. Sampling strategy adequately described (source of products and selection process reported)
Diet characterization	c2. Diet type, format, species, and relevant characteristics clearly described
Sample preparation and matrix handling	c3. Homogenization described
	c4. Digestion method described
	c5. Composite sampling from multiple packages described
Analytical technique	c6. Analytical method for mineral quantification clearly described and appropriate for the analytes measured
Analytical quality control	c7. Analytical accuracy assessed using CRM, recovery studies, or equivalent validation procedures
	c8. Analysis of duplicates/triplicates (analytical precision)
	c9. Limits of detection and/or quantification reported
Reporting completeness	c10. Results reported with appropriate quantitative detail (individual values or a measure of variability)
	c11. Basis of expression of mineral concentrations clearly described (e.g., DM, AF, or energy basis)

**Table 2:** Macromineral composition of commercial and alternative dog diets reported in the literature. Unless otherwise indicated, weight-based values are expressed as g/100 g dry matter. Values expressed per 1000 kcal of metabolizable energy are underlined. Data are presented as mean ± standard deviation (SD) where available, median (range), or range, either as reported in the original studies or calculated from individual diet values as described in the Methods. Mean values are presented without SD when SD was not reported and could not be validly derived. Values reported on a different basis are specifically identified. Ref.: reference.

Ref.	Type of Diet	Ca	P	Ca:P	Mg	Na	K	Cl
***Conventional diets***								
[18]	Adult dry	1.46 ± 0.326	0.875 ± 0.230	1.69 ± 0.156	0.198 ± 0.0741		0.502 ± 0.151	
[21]	Growth dry	1.57 ± 0.470	1.04 ± 0.170	1.40 ± 0.340	0.160 ± 0.0800	0.370 ± 0.100	0.550 ± 0.120	0.680 ± 0.210
[26]	Wet		3.13 ± 1.03			3.36 ± 0.890		
[28] ‡	Wet	3.70	2.80	1.40	0.300	1.90	2.40	
[29] †	Commercial	1.25 ± 0.290	0.900 ± 0.180			0.570 ± 0.530	0.770 ± 0.140	
[30] †	Dry	0.210 ± 0.100	0.140 ± 0.0400		0.0170 ± 0.0100		0.0840 ± 0.0100	
[38]	Dry	0.490–2.59				0.318–0.705	0.529–1.05	
[54]	Weight-loss	3.04 ± 0.518	2.99 ± 0.799	1.03 ± 0.0960	0.332 ± 0.152	1.20 ± 0.380	2.59 ± 0.680	
***Alternative diets***								
[28]	Commercial homemade-style/RMBD	2.18 ± 1.39	1.89 ± 0.689	1.12 ± 0.678	0.320 ± 0.103	0.870 ± 0.604	2.24 ± 0.920	
[33] *	Commercial plant-based: adult	1.07 (0.380–1.90)	0.800 (0.500–1.50)	1.30 (0.800–2.50)	0.150 (0.100–0.230)	0.340 (0.0200–1.27)	1.11 (0.730–1.72)	0.930 (0.0900–2.47)
	Commercial plant-based: growth	1.12 (0.480–1.45)	0.880 (0.580–1.12)	1.30 (0.800–1.40)	0.180 (0.100–0.670)	0.360 (0.290–0.690)	1.21 (0.730–1.72)	1.12 (0.230–1.41)
[57]	Home-prepared, not supplemented	0.310 ± 0.310	1.16 ± 0.770	0.260 ± 0.220	0.250 ± 0.110	0.350 ± 0.290	0.770 ± 0.330	
	Home-prepared, supplemented	1.77 ± 1.52	1.09 ± 0.610	1.78 ± 1.46	0.190 ± 0.110	0.510 ± 0.490	0.850 ± 0.460	
[57]	Home-prepared, with meat	0.810 ± 1.04	1.13 ± 0.450	0.840 ± 1.22	0.210 ± 0.110	0.420 ± 0.370	0.800 ± 0.410	
	Home-prepared, vegetarian	1.38 ± 1.85	1.14 ± 0.410	1.09 ± 1.16	0.280 ± 0.110	0.440 ± 0.480	0.830 ± 0.250	
[60] *	Commercial insect-based	1.30 (0.910–2.81)	0.930 (0.690–1.70)	1.59 (0.580–2.95)	0.210 (0.120–0.310)			
[69] ‡	Home-prepared (growth)	0.0900	0.370	0.250	0.0900	0.110	0.760	
[70] ‡	Home-prepared RMBD with commercial premix (growth)	0.0800	0.440	0.182	0.130	0.105	0.590	
[71]	Commercial RMBD: adult	3.70 ± 2.80	2.30 ± 1.30	1.50 ± 0.600				
	Commercial RMBD: growth	2.70 ± 0.800	1.80 ± 0.200	1.50 ± 0.500				
	Commercial RMBD: all life stages	4.50 ± 3.00	2.20 ± 0.900	1.90 ± 0.700				
[73]	Commercial vegan	0.800 ± 0.220	1.00 ± 0.0600	0.800 ± 0.180	0.170 ± 0.0600	0.270 ± 0.180	0.770 ± 0.450	
***Comparative studies: conventional* vs. *alternative diets***								
[25]	Commercial meat-based	4.68 ± 2.93	3.16 ± 1.19	1.41 ± 0.380	0.320 ± 0.0500	1.05 ± 0.470	1.76 ± 0.390	1.80 ± 1.00
	Commercial plant-based	2.81 ± 1.13	2.24 ± 0.530	1.33 ± 0.630	0.500 ± 0.160	0.910 ± 0.250	2.54 ± 0.470	1.60 ± 0.850
	Commercial renal	1.06 ± 0.240	0.770 ± 0.0800	1.40 ± 0.250	0.220 ± 0.0200	0.560 ± 0.110	1.62 ± 0.100	1.60 ± 0.340
***Comparisons within conventional diets***								
[27] †	Adult	1.02 ± 0.240	0.840 ± 0.290		0.130 ± 0.0300	0.390 ± 0.0700	0.670 ± 0.110	
	Puppy	1.62 ± 0.520	1.15 ± 0.320		0.120 ± 0.0700	0.510 ± 0.140	0.680 ± 0.150	
[31] *	Wet	1.98	1.57		0.135	1.02	1.49	
	Dry	1.29	0.956		0.121	0.409	0.625	
[45]	Cereal-containing dry	1.09 ± 0.308	0.750 ± 0.136	1.47 ± 0.387	0.144 ± 0.0317	0.428 ± 0.0776	0.713 ± 0.127	
	Cereal-free dry	1.08 ± 0.403	0.771 ± 0.194	1.46 ± 0.540	0.145 ± 0.0333	0.527 ± 0.156	0.964 ± 0.161	
[48]	Fish dry	0.455 ± 0.0720	1.34 ± 0.300	0.351 ± 0.0860	0.211 ± 0.0870	0.588 ± 0.190	0.935 ± 0.149	
	Poultry dry	0.449 ± 0.0970	1.31 ± 0.130	0.344 ± 0.0630	0.153 ± 0.0410	0.422 ± 0.0700	0.768 ± 0.137	
[48]	Lamb dry	0.620 ± 0.122	1.36 ± 0.250	0.472 ± 0.147	0.228 ± 0.102	0.443 ± 0.0550	0.739 ± 0.197	
	Beef dry	0.441 ± 0.0400	1.06 ± 0.270	0.443 ± 0.144	0.133 ± 0.0330	0.425 ± 0.0960	0.852 ± 0.124	
	Mixture dry	0.425 ± 0.207	1.27 ± 0.240	0.323 ± 0.141	0.159 ± 0.0490	0.458 ± 0.118	0.871 ± 0.221	
	Other dry	0.450 ± 0.0700	1.26 ± 0.340	0.373 ± 0.114	0.154 ± 0.130	0.683 ± 0.414	0.970 ± 0.142	
[55]	Non-prescription dry	1.07 ± 0.185	0.553 ± 0.107					
	Hepatic dry	0.478 ± 0.226	0.246 ± 0.167					
	Renal dry	0.384 ± 0.102	0.263 ± 0.169					
	Gastrointestinal dry	0.594 ± 0.141	0.345 ± 0.106					
	Hypoallergenic dry	0.658 ± 0.136	0.390 ± 0.124					
[56] †	Dry	1.41 ± 0.120	1.58 ± 0.510		0.160 ± 0.0130			
	Wet	0.680 ± 0.290	1.39 ± 0.290		0.0680 ± 0.0110			
[43]	Super-premium dry	1.02	0.880					
	Premium dry	1.41	1.20					
	White brand 1 dry	2.02	1.51					
	White brand 2 dry	1.40	0.960					
[58]	Low-price dry	2.82	1.48	1.92	0.197	0.724	0.621	
	Medium dry	1.75	1.15	1.52	0.131	0.802	0.740	
	Premium dry	1.85	1.12	1.71	0.163	0.634	0.618	
	Super-premium dry	1.26	0.904	1.41	0.124	0.591	1.00	
[61]	Dry	1.08 ± 0.340	1.00 ± 0.400	1.10 ± 0.300	0.100 ± 0.0300	0.320 ± 0.190	0.460 ± 0.140	
	Wet	2.11 ± 1.19	1.78 ± 0.910	1.20 ± 0.300	0.0900 ± 0.0200	1.14 ± 0.490	1.07 ± 0.470	
[72] †	Adult	1.28–1.57	1.07–1.16	1.10–1.46				
	Puppies, medium/large breeds	1.33–1.45	0.920–1.04	1.32–1.44				
[72] †	Puppies + reproduction	1.38–1.42	0.940–1.02	1.35–1.51				
	Adults, medium/large breeds	0.960–1.35	0.960–1.11	0.910–1.33				

* Median values. † Source basis not explicitly stated; values were converted to g/100 g of the reported basis and were not assumed to represent dry matter. ‡ Values correspond to a single analysed diet/product.

**Table 3:** Trace element composition of commercial and alternative dog diets reported in the literature. Unless otherwise indicated, weight-based values are expressed as mg/100 g dry matter, with selenium expressed as µg/100 g dry matter. Values expressed per 1000 kcal of metabolizable energy are underlined. Data are presented as mean ± standard deviation (SD) where available, median (range), or range, either as reported in the original studies or calculated from individual diet values as described in the Methods. Mean values are presented without SD when SD was not reported and could not be validly derived. Values reported on a different basis are specifically identified. Ref.: reference.

Ref.	Type of Diet	Co	Cu	Fe	I	Mn	Mo	Se	Zn
***Conventional diets***									
[18]	Adult dry		0.981 ± 0.155	28.0 ± 18.5		3.78 ± 4.25		45.0 ± 25.9	9.86 ± 5.36
[21]	Growth dry		3.23 ± 1.09	36.8 ± 12.2	0.415 ± 0.613	5.93 ± 3.20		57.0 ± 27.0	17.9 ± 5.76
[30] †	Dry		1.42 ± 0.700	23.0 ± 6.60		3.70 ± 2.60			20.0 ± 11.4
[38]	Dry	0.00800–0.0590		18.8–64.6				24.0–214	4.40–63.3
[40] *	Dry		4.40 (2.30–9.00)	62.0 (25.5–278)		11.7 (2.30–26.5)		180 (90–490)	53.0 (33.0–82.2)
[41]	Wet				0.0700–5.04				
[42]	Dry & wet	0.0419 ± 0.0678	2.18 ± 1.28				0.0979 ± 0.0449		25.4 ± 15.5
[44]	Dry	0.401 ± 0.133	3.56 ± 1.60	53.0 ± 30.9					2.52 ± 6.82
[46] *	Dry						0.250 (0.0400–0.650)		
[54]	Weight-loss dry		19.5 ± 6.54	99.8 ± 36.0		23.3 ± 11.4		56.2 ± 147	65.9 ± 11.2
[63]	Dry & wet							40.0	
[74]	Dry & wet	0.165 ± 0.236		33.9 ± 21.3					
[28] ‡	Wet		5.00	55.8		4.90		0	5.40
***Alternative diets***									
[28]	Commercial homemade-style/RMBD		2.20 ± 1.50	28.2 ± 17.5		4.64 ± 2.62		30.0 ± 94.9	32.8 ± 33.3
[33] *	Commercial plant-based: adult		2.34 (0.890–5.47)	22.7 (10.7–75.0)		4.13 (1.39–7.39)			15.5 (4.46–35.9)
	Commercial plant-based: growth		3.25 (1.43–3.49)	41.5 (12.7–61.6)		4.83 (1.39–8.26)			15.6 (9.32–35.9)
[57]	Home-prepared, not supplemented		7.30 ± 6.23	9.59 ± 5.02		3.23 ± 2.69		1.53 ± 7.02	11.1 ± 6.93
[57]	Home-prepared, supplemented		11.6 ± 12.4	15.3 ± 12.2		3.96 ± 4.09		0.00 ± 0.00	15.0 ± 10.9
	Home-prepared, with meat		8.98 ± 9.75	12.1 ± 9.90		3.31 ± 3.45		0.580 ± 4.51	13.9 ± 9.43
	Home-prepared, vegetarian/vegan		9.57 ± 8.29	11.1 ± 4.58		4.62 ± 2.62		2.42 ± 8.39	7.27 ± 2.92
[60] *	Commercial insect-based		1.90 (0.641–2.98)	50.8 (7.98–88.9)					25.5 (4.25–51.2)
[70] ‡	Home-prepared RMBD with commercial premix (growth)		0.500	6.20		3.10			4.80
[71]	Commercial RMBD: adult		1.70 ± 0.900						32.6 ± 28.1
	Commercial RMBD: growth		1.60 ± 0.300						37.7 ± 24.8
	Commercial RMBD: all life stages		2.70 ± 2.90						14.9 ± 5.70
[73]	Commercial vegan		4.80 ± 1.61	51.3 ± 20.5		7.37 ± 2.15			21.0 ± 3.00
***Comparative studies: conventional* vs. *alternative diets***									
[25]	Commercial meat-based		4.55 ± 2.25	84.3 ± 57.3	0.280 ± 0.170	12.4 ± 4.50		101 ± 33.0	35.8 ± 11.6
	Commercial plant-based		5.17 ± 1.47	117 ± 99.0	0.140 ± 0.100	17.1 ± 8.20		77.3 ± 25.1	34.3 ± 12.8
	Commercial renal		3.62 ± 1.01	34.1 ± 9.30	0.300 ± 0.0900	13.4 ± 4.20		60.8 ± 25.3	37.2 ± 10.7
***Comparisons within conventional diets***									
[22] †	Dry	0.0316 ± 0.0266					0.0847 ± 0.0399		
	Wet	0.00929 ± 0.0108					0.0246 ± 0.0147		
[27] †	Adult		1.14 ± 0.162	22.1 ± 4.90	70.5 ± 19.4	3.22 ± 1.80			20.1 ± 4.60
	Puppy		3.01 ± 3.60	37.8 ± 10.4	94.2 ± 48.5	4.33 ± 1.50			47.6 ± 55.7
[31] *	Wet	0.0000100				0.00233	<0.0000640 (LOQ)		0.0170
	Dry	0.00000800				0.00579	<0.0000640 (LOQ)		0.0180
[32] †	Adult	0.00800–0.0820	0.900–6.00	17.7–86.2	0.0900–1.40	0.600–11.1			4.40–55.4
	Puppy	0.00800–0.0750	1.50–6.40	19.8–67.5	0.0700–1.40	1.60–14.9			4.60–61.4
[36]	Dry		0.540 ± 0.210	3.84 ± 1.29		0.720 ± 0.370			
	Wet		0.790 ± 0.270	6.52 ± 0.550		1.16 ± 0.620			
[45]	Cereal-containing dry		1.68 ± 0.280	39.9 ± 16.7		4.27 ± 2.63			17.4 ± 4.86
	Cereal-free dry		2.16 ± 0.440	46.9 ± 22.9		5.78 ± 5.66			19.9 ± 8.47
[48]	Fish dry		1.34 ± 0.590	17.7 ± 8.40		2.98 ± 1.50	0.113 ± 0.0250		8.63 ± 3.18
	Poultry dry		1.13 ± 0.140	16.8 ± 5.82		1.80 ± 0.740	0.0980 ± 0.00800		9.51 ± 1.90
	Lamb dry		1.49 ± 0.310	20.9 ± 4.69		3.57 ± 0.450	0.110 ± 0.0140		9.06 ± 3.56
	Beef dry		1.84 ± 0.740	17.4 ± 6.06		3.52 ± 1.53	0.105 ± 0.00800		7.92 ± 2.18
	Mixture dry		1.14 ± 0.280	23.7 ± 12.9		2.43 ± 1.03	0.101 ± 0.0310		10.6 ± 7.36
	Other dry		0.878 ± 0.782	11.9 ± 3.01		2.99 ± 3.09	0.110 ± 0.0170		5.52 ± 3.38
[51] †	Premium dry		1.34 ± 0.230	12.2 ± 2.24		4.59 ± 1.91	0.0406 ± 0.00869	66.0 ± 29.9	17.5 ± 2.52
	Low-cost dry		1.49 ± 0.160	17.7 ± 6.47		4.66 ± 1.40	0.0455 ± 0.0143	71.0 ± 20.6	14.6 ± 2.36
[52]	Junior dry		2.30 ± 0.638	67.8 ± 27.1					31.4 ± 10.8
	Adult dry		4.29 ± 0.874	39.3 ± 12.8					33.2 ± 5.77
[55]	Non-prescription dry		0.944 ± 0.598	49.5 ± 24.7		2.18 ± 1.01		201 ± 210	8.94 ± 6.02
	Hepatic dry		0.868 ± 0.715	20.2 ± 21.0		4.55 ± 6.58		229 ± 368	9.98 ± 3.44
	Renal dry		0.903 ± 0.633	47.9 ± 108		2.25 ± 1.08		101 ± 99.5	11.2 ± 7.03
	Gastrointestinal dry		1.24 ± 0.560	49.9 ± 53.0		3.17 ± 1.21		189 ± 168	12.6 ± 4.93
	Hypoallergenic dry		1.33 ± 0.535	47.1 ± 80.0		3.50 ± 1.87		184 ± 202	16.8 ± 5.95
[56] †	Dry	0.0140 ± 0.00200	1.58 ± 0.120	36.2 ± 4.26		3.78 ± 0.786	0.0870 ± 0.00600	56.0 ± 5.00	13.3 ± 0.879
	Wet	0.0120 ± 0.00800	2.57 ± 1.54	91.0 ± 54.4		2.32 ± 1.28	0.110 ± 0.0250	150 ± 19.0	23.5 ± 6.60
[58]	Low-price dry		2.13	33.2		7.48		43.2	27.9
	Medium dry		1.98	15.2		8.29		52.7	30.6
	Premium dry		1.80	20.8		6.53		102	30.1
[58]	Super-premium dry		2.94	33.6		8.93		95.4	39.8
[61]	Dry	0.140 ± 0.130	1.50 ± 0.740	16.5 ± 10.7		5.35 ± 1.71		6.22 ± 4.05	23.1 ± 15.7
	Wet	0.0400 ± 0.0300	0.850 ± 0.580	17.0 ± 11.6		4.94 ± 4.95		10.6 ± 9.30	11.1 ± 5.95

* Median values. † Source basis not explicitly stated; values were converted to mg/100 g of the reported basis (Se to µg/100 g) and were not assumed to represent dry matter. ‡ Values correspond to a single analysed diet/product.

**Table 4:** Macromineral composition of commercial and alternative cat diets reported in the literature. Unless otherwise indicated, weight-based values are expressed as g/100 g dry matter. Values expressed per 1000 kcal of metabolizable energy are underlined. Data are presented as mean ± standard deviation (SD) where available, median (range), or range, either as reported in the original studies or calculated from individual diet values as described in the Methods. Mean values are presented without SD when SD was not reported and could not be validly derived. Values reported on a different basis are specifically identified. Ref.: reference.

Ref.	Type of Diet	Ca	P	Ca:P	Mg	Na	K	Cl
***Conventional diets***								
[26]	Wet		3.09 ± 1.28			2.76 ± 1.48		
[29] †	Commercial	1.64 ± 0.500	1.18 ± 0.250			0.680 ± 0.780	0.820 ± 0.130	
[30] †	Dry	0.170 ± 0.0660	0.140 ± 0.0290		0.0170 ± 0.00670		0.0880 ± 0.0180	
[72] †	Adult	1.45 ± 0.400	1.07 ± 0.110	1.33 ± 0.250				
***Alternative diets***								
[33] *	Commercial plant-based: adult	0.840 (0.480–1.42)	0.790 (0.560–1.07)	1.10 (0.800–1.40)	0.170 (0.0900–0.190)	0.330 (0.170–0.730)	1.06 (0.680–1.72)	0.950 (0.380–1.46)
	Commercial plant-based: growth	0.960 (0.480–1.39)	0.760 (0.560–1.07)	1.24 (0.830–1.39)	0.180 (0.0900–0.190)	0.330 (0.290–0.730)	1.44 (0.680–1.72)	1.03 (0.790–1.46)
[57]	Home-prepared, not supplemented	0.700 ± 0.700	1.29 ± 0.390	0.500 ± 0.380	0.270 ± 0.110	0.460 ± 0.360	1.00 ± 0.520	
	Home-prepared, supplemented	2.26 ± 1.22	1.33 ± 0.510	2.26 ± 2.19	0.230 ± 0.0700	0.780 ± 0.410	1.07 ± 0.360	
	Home-prepared, with meat	1.76 ± 1.33	1.34 ± 0.470	1.69 ± 2.01	0.230 ± 0.0600	0.700 ± 0.420	1.01 ± 0.370	
	Home-prepared, vegetarian	0.700 ± 0.320	1.13 ± 0.350	0.600 ± 0.0800	0.340 ± 0.190	0.300 ± 0.200	1.30 ± 0.670	
[71]	Commercial RMBD	4.80 ± 2.40	2.90 ± 1.10	1.60 ± 0.300				
[73] ‡	Commercial vegan	0.720	1.07	0.670	0.170	0.200	0.540	
***Comparative studies: conventional* vs. *alternative diets***								
[67] *	Dry	3.90 (1.80–7.40)	3.10 (1.80–4.90)	1.30 (0.800–1.60)	0.300 (0.100–0.500)			
	Wet	3.30 (0.300–8.70)	2.80 (0.600–5.80)	1.30 (0.500–1.70)	0.200 (0.0500–0.490)			
	Commercial raw	4.60 (1.00–8.20)	3.10 (1.20–5.20)	1.40 (0.800–1.60)	0.300 (0.100–0.500)			
***Comparisons within conventional diets***								
[23]	Renal dry	0.615	0.386	1.59	0.0710			
	Urinary dry	0.569	0.436	1.31	0.0710			
	Obesity dry	0.572	0.370	1.55	0.0570			
	Non-prescription dry	1.13	0.606	1.86	0.0920			
	Gastrointestinal dry	0.839	0.603	1.39	0.0930			
[27] †	Adult	1.32 ± 0.370	0.880 ± 0.130		0.140 ± 0.0300	0.530 ± 0.140	0.680 ± 0.180	
	Kitten	1.51 ± 0.310	1.00 ± 0.250		0.140 ± 0.0300	0.500 ± 0.0700	0.580 ± 0.150	
[31] *	Wet	1.74	1.57		0.105	1.19	1.34	
	Dry	1.41	1.24		0.111	0.569	0.769	
[56] †	Dry	1.56 ± 0.750	1.89 ± 0.720		0.150 ± 0.0570			
	Wet	0.700 ± 0.240	1.36 ± 0.310		0.0740 ± 0.0200			
[66] *	Senior-labelled	3.70 (1.90–5.70)	3.20 (1.50–4.40)	1.30 (0.900–1.60)	0.280 (0.120–0.560)	1.10 (0.700–2.70)	2.30 (1.70–3.60)	
	Adult-labelled	3.90 (0.800–8.70)	3.10 (0.900–5.80)	1.30 (0.800–1.70)	0.270 (0.0600–0.540)	1.10 (0.500–3.90)	2.20 (1.20–3.90)	

* Median values. † Source basis not explicitly stated; values were converted to mg/100 g of the reported basis (Se to µg/100 g) and were not assumed to represent dry matter. ‡ Values correspond to a single analysed diet/product.

**Table 5:** Trace element composition of commercial and alternative cat diets reported in the literature. Unless otherwise indicated, weight-based values are expressed as mg/100 g dry matter, with selenium expressed as µg/100 g dry matter. Values expressed per 1000 kcal of metabolizable energy are underlined. Data are presented as mean ± standard deviation (SD) where available, median (range), or range, either as reported in the original studies or calculated from individual diet values as described in the Methods. Mean values are presented without SD when SD was not reported and could not be validly derived. Values reported on a different basis are specifically identified. Ref.: reference.

Ref.	Type of Diet	Co	Cu	Fe	I	Mn	Mo	Se	Zn
***Conventional diets***									
[30] †	Dry		1.50 ± 0.600	27.2 ± 12.3		3.77 ± 2.50			15.5 ± 9.40
[42]	Dry & wet	0.0258 ± 0.0408	1.32 ± 1.34				0.0766 ± 0.0593		13.7 ± 15.7
[44]	Dry	0.244 ± 0.0560	3.81 ± 1.38	38.9 ± 8.85					19.5 ± 5.46
[63]	Dry & wet							114	
[74]	Dry & wet	0.0660 ± 0.0690		38.3 ± 34.5					
***Alternative diets***									
[33] *	Commercial plant-based: adult		2.49 (1.43–3.44)	26.5 (10.5–42.2)		4.11 (1.31–6.72)			18.7 (8.90–35.9)
	Commercial plant-based: growth		2.65 (1.43–3.44)	24.6 (10.5–41.4)		4.10 (1.31–5.55)			21.5 (8.90–35.9)
[57]	Home-prepared, not supplemented		7.23 ± 8.21	10.2 ± 3.47		3.63 ± 4.98		20.7 ± 62.1	10.3 ± 5.52
	Home-prepared, supplemented		15.5 ± 12.9	15.5 ± 5.55		1.65 ± 3.14		0.00	16.5 ± 8.19
	Home-prepared, with meat		12.3 ± 11.9	13.5 ± 5.61		1.58 ± 2.69		0.00	16.5 ± 8.67
	Home-prepared, vegetarian		11.5 ± 12.4	12.7 ± 4.43		8.77 ± 6.41		69.0 ± 97.6	10.9 ± 5.32
[71]	Commercial RMBD: adult		2.60 ± 1.20						27.1 ± 10.7
[73] ‡	Commercial vegan		7.60	39.0		7.00			24.0
***Comparative studies: conventional* vs. *alternative diets***									
[68] *	Dry		6.00 (3.20–10.5)	61.4 (29.8–104)					57.0 (17.2–122)
	Wet		5.20 (1.40–13.3)	59.3 (24.5–379)					43.8 (21.3–128)
	Raw		3.60 (0.800–8.10)	34.5 (15.7–74.4)					18.7 (7.60–43.2)
	Adult-labelled		5.60 (0.800–13.3)	54.8 (15.7–135)					43.6 (7.60–106)
	Senior-labelled		5.70 (3.10–10.5)	64.9 (29.8–379)					57.2 (35.6–128)
***Comparisons within conventional diets***									
[19] *	Wet				0.440 (0.0700–1.94)				
	Dry				0.300 (0.0900–0.910)				
[20] #	Salmon-based wet		0.218 ± 0.123	10.6 ± 7.82		0.246 ± 0.0633			2.08 ± 0.443
	Tuna-based wet		0.295 ± 0.193	13.2 ± 9.57		0.280 ± 0.123			2.02 ± 0.642
	Liver-based wet		0.204 ± 0.168	7.14 ± 5.39		0.364 ± 0.0980			2.14 ± 0.231
	Aquatic-product wet, kitten		0.266 ± 0.233	12.1 ± 12.3		0.264 ± 0.139			2.11 ± 0.635
[22] †	Dry	0.0292 ± 0.0248					0.0968 ± 0.0539		
	Wet	0.00843 ± 0.00953					0.0182 ± 0.0145		
[23]	Renal dry		1.18	68.6		2.41		135	12.3
	Urinary dry		1.53	59.4		3.43		304	14.2
	Obesity dry		1.54	31.2		3.22		176	13.6
	Non-prescription dry		1.06	49.6		1.77		228	7.26
	Gastrointestinal dry		1.51	35.9		2.80		217	15.6
[27] †	Adult		1.07 ± 0.238	26.9 ± 12.7	30.8 ± 9.70	4.81 ± 2.80			18.5 ± 5.80
	Kitten		1.38 ± 0.388	80.9 ± 4.88	29.5 ± 16.5	4.43 ± 1.96			20.9 ± 9.97
[31] *	Wet	0.00000800				0.00197	0.0000750		0.0154
	Dry	0.00000800				0.00596	<0.0000640 (LOQ)		0.0176
[36]	Dry		1.18 ± 0.510	4.26 ± 1.84		1.61 ± 1.13			
	Wet		1.24 ± 0.450	6.88 ± 0.230		1.45 ± 0.820			
[37] *	Dry & wet				0.350 (0.0200–15.5)				
	Kitten dry & wet				0.501 (0.294–0.728)				
	Adult non-therapeutic dry & wet				0.364 (0.0200–15.5)				
	Therapeutic dry & wet				0.261 (0.0840–0.500)				
[51] †	Premium dry		1.34 ± 0.320	13.1 ± 2.42		4.51 ± 2.44	0.0462 ± 0.0308	76.1 ± 15.4	17.7 ± 3.01
	Low-cost dry		1.49 ± 0.390	19.0 ± 4.34		4.22 ± 1.57	0.0427 ± 0.0177	77.5 ± 18.7	16.1 ± 4.38
[56] †	Dry	0.0130 ± 0.00600	1.65 ± 0.380	38.4 ± 10.2		2.59 ± 1.25	0.0880 ± 0.0380	74.0 ± 36.0	16.7 ± 6.40
	Wet	0.0100 ± 0.00500	2.01 ± 1.20	89.6 ± 61.8		3.73 ± 1.61	0.0800 ± 0.0300	120 ± 43.0	15.8 ± 6.70

* Median values. † Source basis not explicitly stated; values were converted to mg/100 g of the reported basis (Se to µg/100 g) and were not assumed to represent dry matter. ‡ Values correspond to a single analysed diet/product. # Values are expressed on a wet-weight basis.

**Table 6:** Coefficients of variation (CV, %) for mineral concentrations in dog and cat diets included in the analytical synthesis.

Ref.	Type of Diet	Ca	P	Ca:P	Mg	Na	K	Cl	Co	Cu	Fe	I	Mn	Mo	Se	Zn
***Conventional diets***																
[18]	Adult dry dog	22	26	9	37		30			16	66		112		58	54
[21]	Growth dry dog	30	16	24	50	27	22	31		34	33	148	54		47	32
[26]	Wet dog		33			26										
[29]	Commercial dog	23	20			93	18									
[30]	Dry dog	48	29		59		12			49	29		70			57
[42]	Dry & wet dog								162	59				46		61
[44]	Dry dog								33	45	58					271
[54]	Weight-loss dry dog	17	27	9	46	32	26			34	36		49		262	17
[74]	Dry & wet dog								143		63					
[26]	Wet cat		41			54										
[29]	Commercial cat	30	21			115	16									
[30]	Dry cat	39	21		39		20			40	45		66			61
[42]	Dry & wet cat								158	102				77		115
[44]	Dry cat								23	36	23					28
[72]	Adult cat	28	10	19												
[74]	Dry & wet cat								105		90					
***Alternative diets***																
[28]	Commercial homemade-style/RMBD	64	36	61	32	69	41			68	62		56		316	102
[57]	Home-prepared, not supplemented dog	100	66	85	44	83	43			85	52		83		459	62
	Home-prepared, supplemented dog	86	56	82	58	96	54			107	80		103			73
	Home-prepared, with meat dog	128	40	145	52	88	51			109	82		104		778	68
	Home-prepared, vegetarian dog	134	36	106	39	109	30			87	41		57		347	40
[71]	Commercial RMBD: adult dog	76	57	40						53						86
	Commercial RMBD: growth dog	30	11	33						19						66
	Commercial RMBD: all life stages dog	67	41	37						107						38
[73]	Commercial vegan dog	27	6	22	35	67	58			34	40		29			14
[57]	Home-prepared, not supplemented cat	100	30	76	41	78	52			114	34		137		300	54
	Home-prepared, supplemented cat	54	38	97	30	53	34			83	36		190			50
	Home-prepared, with meat cat	76	35	119	26	60	37			97	42		170			53
	Home-prepared, vegetarian cat	46	31	13	56	67	52			108	35		73		141	49
[71]	Commercial RMBD cat	50	38	19												
	Commercial RMBD: adult cat									46						39
***Comparative studies: conventional* vs. *alternative diets***																
[25]	Commercial meat-based dog	63	38	27	16	45	22	56		49	68	61	36		33	32
	Commercial plant-based dog	40	24	47	32	27	19	53		28	85	71	48		32	37
	Commercial renal dog	23	10	18	9	20	6	21		28	27	30	31		42	29
***Comparisons within conventional diets***																
[22]	Dry dog								84					47		
	Wet dog								116					60		
[27]	Adult dry dog	24	35		23	18	16			14	22	28	56			23
	Puppy dry dog	32	28		58	27	22			120	28	51	35			117
[36]	Dry dog									39	34		51			
	Wet dog									34	8		53			
[45]	Cereal-containing dry dog	28	18	26	22	18	18			17	42		62			28
	Cereal-free dry dog	37	25	37	23	30	17			20	49		98			43
[48]	Fish dry dog	16	22	25	41	32	16			44	47		50	22		37
	Poultry dry dog	22	10	18	27	17	18			12	35		41	8		20
	Lamb dry dog	20	18	31	45	12	27			21	22		13	13		39
	Beef dry dog	9	25	33	25	23	15			40	35		43	8		28
	Mixture dry dog	49	19	44	31	26	25			25	54		42	31		69
	Other dry dog	16	27	31	84	61	15			89	25		103	15		61
[51]	Premium dry dog									17	18		42	21	45	14
	Low-cost dry dog									11	37		30	31	29	16
[52]	Junior dry dog									28	40					34
	Adult dry dog									20	33					17
[55]	Non-prescription dry dog	17	19							63	50		46		104	67
	Hepatic dry dog	47	68							82	104		145		161	34
	Renal dry dog	27	64							70	225		48		99	63
	Gastrointestinal dry dog	24	31							45	106		38		89	39
	Hypoallergenic dry dog	21	32							40	170		53		110	35
[56]	Dry dog	9	32		8				14	8	12		21	7	9	7
	Wet dog	43	21		16				67	60	60		55	23	13	28
[61]	Dry dog	31	40	27	30	59	30		93	49	65		32		65	68
	Wet dog	56	51	25	22	43	44		75	68	68		100		88	54
[20]	Salmon-based wet cat									56	74		26			21
	Tuna-based wet cat									65	73		44			32
	Liver-based wet cat									82	75		27			11
	Aquatic-product, wet kitten									88	102		53			30
[22]	Dry cat								85					56		
	Wet cat								113					80		
[27]	Adult dry cat	28	15		21	26	26			22	47	31	58			31
	Kitten dry cat	21	25		21	14	26			28	6	56	44			48
[36]	Dry cat									43	43		70			
	Wet cat									36	3		57			
[51]	Premium dry cat									24	18		54	67	20	17
	Low-cost dry cat									26	23		37	41	24	27
[56]	Dry cat	48	38		38				46	23	27		48	43	49	38
	Wet cat	34	23		27				50	60	69		43	38	36	42

## Data Availability

The data that support the review findings are available from the corresponding author upon request.

## Supplementary Materials

The following supporting information can be downloaded at https://www.mdpi.com/article/10.3390/ani16203261/s1, Supplementary File S1: Database search strategies; Table S1: Overview of analysis-based studies assessing mineral intake or adequacy in dog and cat diets; Table S2: Overview of estimated-based assessing intake or adequacy in dogs and cat diets; Table S3: Minimum and maximum recommended macro- and micromineral levels for dogs according to FEDIAF and AAFCO guidelines.; Table S4: Minimum and maximum recommended macro- and micromineral levels for cats according to FEDIAF and AAFCO guidelines; Table S5: Methodological and analytical quality assessment of studies reporting mineral concentrations in complete dog and cat diets.

## Abbreviations

The following abbreviations are used in this manuscript:

AAFCO	Association of American Feed Control Officials
Ca:P	Calcium-to-phosphorus ratio
FEDIAF	European Pet Food Industry Federation
RMBDs	Raw meat-based diets
SD	Standard deviation
