# Supplementary material for: Mineral Composition and Adequacy of Mineral Concentrations in Dog and Cat Diets: A Systematic Review
Source: Animals (Basel). 2026 Oct 16;16(20):3261. doi: 10.3390/ani16203261 (PMC13700775; doi:10.3390/ani16203261)
Supplement: Supplementary file 1 [file animals-16-03261-s001.zip › animals-4491318-supplementary.pdf]

SUPPLEMENTARY FILE S1: SEARCH STRATEGIES AND SUPPLEMENTARY TABLES

Complete database search strategies

PubMed:

- Search A: Mineral composition of diets and foods: (dog[Title/Abstract] OR dogs[Title/Abstract] OR canine*[Title/Abstract] OR cat[Title/Abstract] OR cats[Title/Abstract] OR feline*[Title/Abstract]) AND ("pet food"[Title/Abstract] OR "pet foods"[Title/Abstract] OR "dog food"[Title/Abstract] OR "dog foods"[Title/Abstract] OR dogfood*[Title/Abstract] OR "cat food"[Title/Abstract] OR "cat foods"[Title/Abstract] OR ration*[Title/Abstract] OR recipe*[Title/Abstract] OR ((commercial[Title/Abstract] OR complete[Title/Abstract] OR dry[Title/Abstract] OR wet[Title/Abstract] OR canned[Title/Abstract] OR raw[Title/Abstract] OR homemade[Title/Abstract] OR "home-prepared"[Title/Abstract] OR vegan[Title/Abstract] OR vegetarian[Title/Abstract] OR therapeutic[Title/Abstract] OR prescription[Title/Abstract]) AND (diet*[Title/Abstract] OR food*[Title/Abstract]))) AND (mineral*[Title/Abstract] OR "trace element"[Title/Abstract] OR "trace elements"[Title/Abstract] OR micromineral*[Title/Abstract] OR macromineral*[Title/Abstract] OR "heavy metal"[Title/Abstract] OR "heavy metals"[Title/Abstract] OR "potentially toxic element"[Title/Abstract] OR "potentially toxic elements"[Title/Abstract] OR "chemical composition"[Title/Abstract] OR "elemental composition"[Title/Abstract] OR ((calcium[Title/Abstract] OR phosphorus[Title/Abstract] OR magnesium[Title/Abstract] OR sodium[Title/Abstract] OR potassium[Title/Abstract] OR chloride[Title/Abstract] OR chlorine[Title/Abstract] OR iron[Title/Abstract] OR zinc[Title/Abstract] OR copper[Title/Abstract] OR iodine[Title/Abstract] OR selenium[Title/Abstract] OR manganese[Title/Abstract] OR cobalt[Title/Abstract] OR molybdenum[Title/Abstract] OR arsenic[Title/Abstract] OR cadmium[Title/Abstract] OR lead[Title/Abstract] OR mercury[Title/Abstract] OR chromium[Title/Abstract] OR nickel[Title/Abstract]) AND (content*[Title/Abstract] OR concentration*[Title/Abstract] OR composition[Title/Abstract] OR level*[Title/Abstract] OR analysis[Title/Abstract] OR adequacy[Title/Abstract] OR intake*[Title/Abstract] OR requirement*[Title/Abstract]))) AND ("2005/01/01"[Date - Publication] : "2025/10/04"[Date - Publication])
- Search B: Nutritional adequacy and nutrient intake: (dog[Title/Abstract] OR dogs[Title/Abstract] OR canine*[Title/Abstract] OR cat[Title/Abstract] OR cats[Title/Abstract] OR feline*[Title/Abstract]) AND (diet[Title/Abstract] OR diets[Title/Abstract] OR dietary[Title/Abstract] OR food[Title/Abstract] OR foods[Title/Abstract] OR feeding[Title/Abstract] OR ration[Title/Abstract] OR rations[Title/Abstract] OR recipe[Title/Abstract] OR recipes[Title/Abstract] OR "pet food"[Title/Abstract] OR "pet foods"[Title/Abstract] OR "dog food"[Title/Abstract] OR "dog foods"[Title/Abstract] OR "cat food"[Title/Abstract] OR "cat foods"[Title/Abstract] OR "weight loss"[Title/Abstract] OR "weight reduction"[Title/Abstract] OR "weight management"[Title/Abstract] OR "energy restriction"[Title/Abstract] OR "caloric restriction"[Title/Abstract]) AND ("nutritional adequacy"[Title/Abstract] OR "nutrient adequacy"[Title/Abstract] OR "nutritional analysis"[Title/Abstract] OR "nutrient analysis"[Title/Abstract] OR "nutritional evaluation"[Title/Abstract] OR "nutrient evaluation"[Title/Abstract] OR "nutrient intake"[Title/Abstract] OR "nutrient intakes"[Title/Abstract] OR "essential nutrient intake"[Title/Abstract] OR "essential nutrient intakes"[Title/Abstract] OR "nutritional deficiency"[Title/Abstract] OR "nutritional deficiencies"[Title/Abstract] OR "nutrient deficiency"[Title/Abstract] OR "nutrient deficiencies"[Title/Abstract] OR "nutrient requirement"[Title/Abstract] OR "nutrient

requirements"[Title/Abstract] OR ((calcium[Title/Abstract] OR phosphorus[Title/Abstract] OR magnesium[Title/Abstract] OR sodium[Title/Abstract] OR potassium[Title/Abstract] OR chloride[Title/Abstract] OR chlorine[Title/Abstract] OR iron[Title/Abstract] OR zinc[Title/Abstract] OR copper[Title/Abstract] OR iodine[Title/Abstract] OR selenium[Title/Abstract] OR manganese[Title/Abstract] OR cobalt[Title/Abstract] OR molybdenum[Title/Abstract]) AND (intake[Title/Abstract] OR intakes[Title/Abstract] OR requirement[Title/Abstract] OR requirements[Title/Abstract] OR deficiency[Title/Abstract] OR deficiencies[Title/Abstract] OR adequacy[Title/Abstract]))) AND ("2005/01/01"[Date - Publication] : "2025/10/04"[Date - Publication])

Scopus:

- Search A: Mineral composition of diets and foods: TITLE-ABS-KEY((dog OR dogs OR canine* OR cat OR cats OR feline*) AND ("pet food" OR "pet foods" OR "dog food" OR "dog foods" OR dogfood* OR "cat food" OR "cat foods" OR ration* OR recipe* OR ((commercial OR complete OR dry OR wet OR canned OR raw OR homemade OR "home-prepared" OR vegan OR vegetarian OR therapeutic OR prescription) AND (diet* OR food*))) AND (mineral* OR "trace element" OR "trace elements" OR micromineral* OR macromineral* OR "heavy metal" OR "heavy metals" OR "potentially toxic element" OR "potentially toxic elements" OR "chemical composition" OR "elemental composition" OR ((calcium OR phosphorus OR magnesium OR sodium OR potassium OR chloride OR chlorine OR iron OR zinc OR copper OR iodine OR selenium OR manganese OR cobalt OR molybdenum OR arsenic OR cadmium OR lead OR mercury OR chromium OR nickel) AND (content* OR concentration* OR composition OR level* OR analysis OR adequacy OR intake* OR requirement*)))) AND PUBYEAR AFT 2004 AND PUBYEAR BEF 2026
- Search B: Nutritional adequacy and mineral/nutrient intake: TITLE-ABS-KEY((dog OR dogs OR canine* OR cat OR cats OR feline*) AND (diet OR diets OR dietary OR food OR foods OR feeding OR ration OR rations OR recipe OR recipes OR "pet food" OR "pet foods" OR "dog food" OR "dog foods" OR "cat food" OR "cat foods" OR "weight loss" OR "weight reduction" OR "weight management" OR "energy restriction" OR "caloric restriction") AND ("nutritional adequacy" OR "nutrient adequacy" OR "nutritional analysis" OR "nutrient analysis" OR "nutritional evaluation" OR "nutrient evaluation" OR "nutrient intake" OR "nutrient intakes" OR "essential nutrient intake" OR "essential nutrient intakes" OR "nutritional deficiency" OR "nutritional deficiencies" OR "nutrient deficiency" OR "nutrient deficiencies" OR "nutrient requirement" OR "nutrient requirements" OR ((calcium OR phosphorus OR magnesium OR sodium OR potassium OR chloride OR chlorine OR iron OR zinc OR copper OR iodine OR selenium OR manganese OR cobalt OR molybdenum) AND (intake OR intakes OR requirement OR requirements OR deficiency OR deficiencies OR adequacy)))) AND PUBYEAR AFT 2004 AND PUBYEAR BEF 2026

Web of Science

- Search A: Mineral composition of diets and foods: TS=((dog OR dogs OR canine* OR cat OR cats OR feline*) AND ("pet food" OR "pet foods" OR "dog food" OR "dog foods" OR dogfood* OR "cat food" OR "cat foods" OR ration* OR recipe* OR ((commercial OR complete OR dry OR wet OR canned OR raw OR homemade OR "home-prepared" OR vegan OR vegetarian OR therapeutic OR prescription) AND (diet* OR food*))) AND (mineral* OR "trace element" OR "trace elements" OR micromineral* OR macromineral* OR "heavy metal" OR "heavy metals" OR "potentially toxic element" OR "potentially toxic elements" OR "chemical composition" OR "elemental composition" OR ((calcium OR phosphorus OR magnesium OR sodium OR potassium OR chloride OR chlorine OR iron OR zinc OR copper OR iodine OR selenium OR manganese OR cobalt OR molybdenum OR arsenic OR cadmium OR lead OR mercury OR chromium OR

nickel) AND (content* OR concentration* OR composition OR level* OR analysis OR adequacy OR intake* OR requirement*)))) AND DOP=2005-01-01/2025-10-04

- Search B: Nutritional adequacy and mineral/nutrient intake: TS=((dog OR dogs OR canine* OR cat OR cats OR feline*) AND (diet OR diets OR dietary OR food OR foods OR feeding OR ration OR rations OR recipe OR recipes OR "pet food" OR "pet foods" OR "dog food" OR "dog foods" OR "cat food" OR "cat foods" OR "weight loss" OR "weight reduction" OR "weight management" OR "energy restriction" OR "caloric restriction") AND ("nutritional adequacy" OR "nutrient adequacy" OR "nutritional analysis" OR "nutrient analysis" OR "nutritional evaluation" OR "nutrient evaluation" OR "nutrient intake" OR "nutrient intakes" OR "essential nutrient intake" OR "essential nutrient intakes" OR "nutritional deficiency" OR "nutritional deficiencies" OR "nutrient deficiency" OR "nutrient deficiencies" OR "nutrient requirement" OR "nutrient requirements" OR ((calcium OR phosphorus OR magnesium OR sodium OR potassium OR chloride OR chlorine OR iron OR zinc OR copper OR iodine OR selenium OR manganese OR cobalt OR molybdenum) AND (intake OR intakes OR requirement OR requirements OR deficiency OR deficiencies OR adequacy)))) AND DOP=2005-01-01/2025-10-04

AGRIS

- Search A: Mineral composition of diets and foods: (dog OR dogs OR canine* OR cat OR cats OR feline*) AND ("pet food" OR "pet foods" OR "dog food" OR "dog foods" OR dogfood* OR "cat food" OR "cat foods" OR ration* OR recipe* OR ((commercial OR complete OR dry OR wet OR canned OR raw OR homemade OR "home-prepared" OR vegan OR vegetarian OR therapeutic OR prescription) AND (diet* OR food*))) AND (mineral* OR "trace element" OR "trace elements" OR micromineral* OR macromineral* OR "heavy metal" OR "heavy metals" OR "potentially toxic element" OR "potentially toxic elements" OR "chemical composition" OR "elemental composition" OR ((calcium OR phosphorus OR magnesium OR sodium OR potassium OR chloride OR chlorine OR iron OR zinc OR copper OR iodine OR selenium OR manganese OR cobalt OR molybdenum OR arsenic OR cadmium OR lead OR mercury OR chromium OR nickel) AND (content* OR concentration* OR composition OR level* OR analysis OR adequacy OR intake* OR requirement*)))
- Search B3 — Nutritional adequacy and mineral/nutrient intake: (dog OR dogs OR canine* OR cat OR cats OR feline*) AND (diet OR diets OR dietary OR food OR foods OR feeding OR ration OR rations OR recipe OR recipes OR "pet food" OR "pet foods" OR "dog food" OR "dog foods" OR "cat food" OR "cat foods" OR "weight loss" OR "weight reduction" OR "weight management" OR "energy restriction" OR "caloric restriction") AND ("nutritional adequacy" OR "nutrient adequacy" OR "nutritional analysis" OR "nutrient analysis" OR "nutritional evaluation" OR "nutrient evaluation" OR "nutrient intake" OR "nutrient intakes" OR "essential nutrient intake" OR "essential nutrient intakes" OR "nutritional deficiency" OR "nutritional deficiencies" OR "nutrient deficiency" OR "nutrient deficiencies" OR "nutrient requirement" OR "nutrient requirements" OR ((calcium OR phosphorus OR magnesium OR sodium OR potassium OR chloride OR chlorine OR iron OR zinc OR copper OR iodine OR selenium OR manganese OR cobalt OR molybdenum) AND (intake OR intakes OR requirement OR requirements OR deficiency OR deficiencies OR adequacy)))

Supplementary Tables S1-S5

Table S1. Summary of analytical studies assessing mineral composition, mineral intake, dietary exposure, or adequacy in complete dog and cat diets.

Reference	Species	Diet category	n	Country	Study purpose
**Abd-Elhakim et al., 2016 [17]**	Dog & cat	Commercial (wet/dry)	20	Egypt	Direct heavy-metal analysis (Cd, Cr, Pb and Sn) of commercial dog and cat foods, including comparisons by species and food format.
**Akinrinmade & Akinrinde, 2011 [18]**	Dog	Commercial (dry)	6	Nigeria	Direct macro- and micromineral composition survey of commercial dry dog foods.
**Alborough et al., 2022 [19]**	Cat	Commercial (wet/dry)	119	United Kingdom	Measures iodine in commercial cat foods, evaluates compliance, habitual exposure and batch-to-batch variation.
**Altınok-Yipel et al., 2022 [20]**	Cat	Commercial (canned/pouched wet)	50	Turkey	Direct essential/toxic metal analysis of commercial canned/pouched cat foods plus dietary exposure/risk assessment.
**Alvarado et al., 2008 [21]**	Dog	Commercial (growth)	26	Chile	To evaluate mineral quality in foods marketed specifically for puppies.
**Atkins et al., 2011 [22]**	Dog & cat	Commercial (dry/wet)	58	USA	Direct ICP-MS trace-element survey of finished commercial pet foods.
**Bilgiç et al., 2025 [23]**	Cat	Prescription vs non-prescription (dry)	96	Turkey	To determine trace/macro elements and to evaluate the potential non-compliance.
**Bilgiç et al., 2026 [24]**	Dog & cat	Prescription vs non-prescription (dry)	236	Turkey	To evaluate potentially toxic elements (As, Al, Cd, Pb, etc.) and chronic risk.
**Brociek et al., 2025 [25]**	Dog	Commercial (plant- vs meat-based)	31	UK	To compare the mineral profiles of plant-based vs meat-based commercial dry foods.
**Brunetto et al., 2019 [26]**	Dog & cat	Commercial (wet)	25	Brazil	Direct Na and P composition with explicit adequacy/excess assessment in commercial wet dog/cat foods.
**Cedeño-López & WingChing-Jones, 2022 [27]**	Dog & cat	Commercial	20	Costa Rica	To determine the multi-mineral profile of imported pet foods and compare it with nutritional recommendations.
**Choi et al., 2023 [28]**	Dog	Commercial homemade style (raw & cooked)	11	Korea	To assess the adequacy of "human-grade" cooked and RMBDs for maintenance.
**Choi et al., 2024 [29]**	Dog & cat	Commercial	55	South Korea	Direct mineral analysis and explicit nutritional-adequacy evaluation of commercial dog/cat foods.
**Costa et al., 2018 [30]**	Dog & cat	Commercial (dry)	41	Brazil	Direct ICP-OES determination of macro-, micro- and trace elements in commercial dry dog and cat foods, with comparison against regulatory recommendations.
**Davies et al., 2017 [31]**	Dog & cat	Commercial (dry & wet)	177	UK	To assess large-scale compliance with FEDIAF nutritional guidelines.

Table S1. Continuation.

Reference	Species	Diet category	n	Country	Study purpose
**De Nadai Fernandes et al., 2018 [32]**	Dog	Commercial (dry)	95	Brazil	Large commercial-food trace-element safety survey with direct multielement analysis.
**Dodd et al., 2021 [33]**	Dog & cat	Commercial (plant based)	26	Canada	To compare commercial plant-based pet foods against guidelines and assess label compliance.
**Du et al., 2025 [34]**	Dog & cat	Commercial (dry/canned)	93	China	Direct analysis of toxic metals in commercial dog/cat foods.
**Dunham-Cheatham et al., 2021 [35]**	Dog & cat	Commercial	111	USA	Direct total-Hg/methylmercury measurements in commercial dog/cat foods; ingredient-DNA work is ancillary.
**Duran et al., 2010 [36]**	Dog & cat	Commercial (dry/wet)	15	Turkey	To determine trace element concentrations and evaluate legal compliance.
**Edinboro et al., 2013 [37]**	Cat	Commercial (canned, pouched & dry)	112	USA	Large direct iodine-composition survey across canned, pouched and dry cat foods with estimated daily iodine exposure.
**Elias et al., 2012 [38]**	Dog	Commercial (dry)	34	Brazil	To evaluate the mineral composition and safety of commercial dry dog foods in Brazil using neutron activation analysis.
**Fantinati et al., 2021 [39]**	Cat	Commercial (plant-based)	1	Italy	Commercial vegan cat food is directly analysed for trace elements and adequacy is assessed against actual intake in affected cats.
**Gagné et al., 2013 [40]**	Dog	Commercial (dry)	50	USA	Direct measurement of Ca, P, Zn, Fe, Cu, Mn and Se in 45 maintenance and 5 therapeutic dry dog foods, with comparison against AAFCO recommendations.
**Guidi et al., 2021 [41]**	Dog & cat	Commercial (canned)	12	Italy	Direct total and soluble iodine measurement in complete canned dog/cat foods with adequacy comparison.
**Haydous et al., 2025 [42]**	Dog & cat	Commercial (dry & wet)	196	Lebanon & Emirates	Large commercial pet-food survey directly measures multiple essential/toxic elements.
**Ivars et al., 2016 [43]**	Dog	Commercial (dry)	4	Spain	Direct physicochemical evaluation of complete commercial dry dog foods, including analytically measured calcium and phosphorus.
**Kalicharan et al., 2025 [44]**	Dog & cat	Commercial (dry)	31	South Africa	Direct multi-element analysis of commercial dog/cat foods, including essential and potentially toxic elements.
**Kazimierska et al., 2020 [45]**	Dog	Commercial (cereal vs cereal-free)	30	Poland	To evaluate and compare mineral profiles and heavy metals in cereal-free vs conventional diets.
**Kim et al., 2018 [46]**	Dog	Commercial (dry)	49	USA	To measure trace element levels in commercial dry dog foods and compare them with WHO human daily intake guidelines.
**Kim et al., 2018 [47]**	Dog	Commercial (dry; poultry, red meat & fish based)	51	USA	Direct toxic-element composition and exposure assessment in complete commercial dog foods.

Table S1. Continuation.

Reference	Species	Diet category	n	Country	Study purpose
**Kępińska-Pacelik et al., 2023 [48]**	Dog	Commercial (dry)	41	Poland	To determine whether the main animal ingredient affects mineral and heavy metal levels.
**Luippold et al., 2016 [49]**	Dog & cat	Commercial (wet/dry)	101	USA	Direct mercury composition survey of commercial cat/dog foods, including ingredient-pattern and lot-variation observations.
**Luís et al., 2023 [50]**	Dog	Commercial weight-loss	5	Brazil	To evaluate actual mineral intake in dogs fed a severe calorie-restricted diet.
**Macías-Montes et al., 2021 [51]**	Dog & cat	Commercial (dry)	61	Spain	Direct ICP-MS analysis of 43 elements in commercial dry dog/cat foods with nutritional and toxic-element risk assessment.
**Mielczarek & Szydłowski, 2017 [52]**	Dog	Commercial (dry; junior & adult)	12	Poland	Direct determination of Cd, Hg, Pb, Fe, Zn and Cu in commercial dry dog foods for junior and adult dogs, with comparison against legal limits.
**Naranjo-Jiménez et al., 2023 [53]**	Dog & cat	Commercial	20	Costa Rica	Direct As/Cd/Hg/Pb analysis in imported commercial pet foods with exposure/adequacy interpretation.
**Olivindo et al., 2022 [54]**	Dog	Commercial weight-loss	8	Brazil	To assess whether mineral density in weight-loss diets is sufficient to maintain adequate levels under caloric restriction.
**Or M.E. et al., 2024 [55]**	Dog	Commercial prescription vs non-prescription (dry)	150	Turkey	Direct measurement of essential minerals in prescription and non-prescription complete dog diets.
**Paulelli et al., 2018 [56]**	Dog & cat	Commercial (dry/canned)	88	Brazil	To perform a toxicological risk assessment for daily element intake.
**Pedrinelli et al., 2021 [57]**	Dog & cat	Home-prepared recipes	100	Brazil	To evaluate the effect of ingredient number, supplementation, and vegan/vegetarian formulation on homemade pet diets.
**Pereira et al., 2018 [58]**	Dog	Commercial (dry)	26	Portugal	To characterize mineral profiles across price segments and assess potential toxicity.
**Prantil et al., 2016 [59]**	Cat	Commercial enteral	4	USA	Seven enteral products were directly analysed; only the four complete/balanced diets are eligible. Published mineral summaries pool all seven products, so this report is retained qualitatively only.
**Ryu et al., 2025 [60]**	Dog	Commercial (insect-based)	18	South Korea	Nutritional profiling of insect-based dog foods includes directly measured minerals as a substantive part of food composition assessment.
**Sgorlon et al., 2022 [61]**	Dog	Dry vs wet	37	Italy	To compare the impact of dietary format on total mineral density.
**Silva et al., 2021 [62]**	Cat	Commercial wet foods	10	Brazil	Direct total-Hg measurement in commercial cat foods plus estimated dietary exposure.
**Simcock et al., 2005 [63]**	Dog & cat	Commercial complete wet & dry	89	New Zealand	Direct fluorometric total-Se survey of complete commercial pet foods with adequacy and format/flavour comparisons.
**Sires et al., 2019 [64]**	Dog	Commercial canine diets	24	USA	Direct toxic-element speciation/composition in commercial dog diets with chronic-exposure assessment.

Table S1. Continuation.

Reference	Species	Diet category	n	Country	Study purpose
**Squadrone et al., 2017 [65]**	Dog & cat	Commercial (fish- vs meat-based)	133	Italy	Direct arsenic surveillance includes a large eligible complete-pet-food subset and regulatory-compliance context.
**Summers et al., 2020 [66]**	Cat	Commercial (senior vs adult)	90	USA	Direct mineral composition and adequacy comparison of commercial senior versus adult cat foods.
**Summers et al., 2020 [67]**	Cat	Commercial (dry/canned/raw)	82	USA	Mineral composition/adequacy is the primary study question, including Ca:P and food-format comparisons.
**Summers et al., 2022 [68]**	Cat	Commercial (dry/canned/raw)	112	USA	Direct Fe/Cu/Zn analysis across 112 commercial cat foods with adequacy comparisons by format and life-stage claims.
**Tal et al., 2018 [69]**	Dog	Home-prepared	1	Canada	Direct laboratory analysis of the complete homemade diet demonstrates multiple mineral deficiencies and an inverted Ca:P ratio central to the clinical case.
**Taylor et al., 2009 [70]**	Dog	Home-prepared (raw meat–based + organic premix)	1	USA	Complete ration as fed was sent to a commercial laboratory; direct mineral analysis documented severe imbalance.
**Vecchiato et al., 2022 [71]**	Dog & cat	Commercial (RMBD/raw)	31	Italy	Direct mineral adequacy assessment of commercial RMBDs explicitly labelled complete.
**Vranić et al., 2023 [72]**	Dog & cat	Commercial	23	Serbia	Direct Ca and P measurement and Ca:P evaluation in commercial dog/cat foods.
**Zafalon et al., 2020 [73]**	Dog & cat	Commercial (vegan)	4	Brazil	To evaluate nutritional adequacy of commercial vegan pet foods and to make comparisons relative to FEDIAF and AAFCO guidelines.
**Zafalon et al., 2021 [74]**	Dog & cat	Commercial (dry & wet)	100	Brazil	Direct toxic-element analysis of 100 complete and balanced commercial dog/cat foods with comparison against tolerable levels.

Table S2. Summary of estimated/adequacy studies assessing mineral intake, dietary exposure, or adequacy in complete dog and cat diets.

Reference	Species	Diet category	n	Country	Study purpose
**Böswald et al., 2018 [75]**	Dog & cat	Mixed feeding histories; CKD vs controls	166	Germany	Estimated long-term P supply and adequacy relative to NRC are primary study variables in dogs and cats with/without CKD.
**de Fornel-Thibaud et al., 2007 [76]**	Dog	Home-prepared	3	France	Calculated mineral composition of homemade diets demonstrates severe Ca/vitamin-D inadequacy and subsequent correction.
**Dillitzer et al., 2011 [77]**	Dog	RMBDs	95	Germany	To evaluate vitamin and mineral supply of owner-fed BARF rations and identify nutritional imbalances relative to NRC recommendations.
**Dodd et al., 2021 [78]**	Dog	Home-prepared raw	1	Canada	Reconstructed complete homemade raw diet demonstrates marked Ca/P inadequacy associated with skeletal disease.
**German et al., 2015 [79]**	Dog	Weight-loss	27	UK	To assess essential-nutrient intake adequacy during long-term controlled weight loss using a purpose-formulated diet.
**German et al., 2023 [80]**	Cat	Weight-loss	58	UK	To compare complete versus partial weight-reduction protocols and assess adequacy of essential nutrient intake during controlled weight loss.
**Hajek et al., 2022 [81]**	Dog	RMBDs	104	Germany	To model nutrient supply of commercial vs owner-formulated recipes via software.
**Hutchinson et al., 2011 [82]**	Dog	Commercial senior diets	37	USA	Manufacturer-provided sodium and phosphorus profiles characterize variability among commercial senior diets.
**Hutchinson et al., 2012 [83]**	Dog	Home-prepared mixed diet	1	USA	Computer reconstruction of the complete homemade diet identifies multiple severe mineral deficiencies.
**Keller et al., 2020 [84]**	Dog & cat	Weight-loss	84	France	To assess short-term weight reduction with reduced-energy maintenance diets and determine whether essential nutrient intakes remain adequate during modest caloric restriction.
**Larsen et al., 2012 [85]**	Dog & cat	Homemade renal	67	USA	To evaluate home-prepared recipes promoted for dogs and cats with CKD, compare their nutrient profiles with NRC recommendations, and assess suitability for CKD management.
**Linder et al., 2012 [86]**	Dog	Weight-loss	5	USA	To determine which nutrients might become inadequate when commercial canine diets are fed under progressively greater caloric restriction.
**McMillan et al., 2006 [87]**	Dog	Veterinary therapeutic renal diet followed by growth diet	2	USA	Quantified Ca/P exposure and Ca:P inadequacy of a renal diet fed during growth are linked to dietary-induced rickets.
**Moscoso et al., 2025 [88]**	Cat	Home-prepared	1	Switzerland	Complete homemade kitten diet is reconstructed and evaluated for Ca/P and other nutrient imbalances linked to nutritional disease and subsequent correction.
**Pedrinelli et al., 2017 [89]**	Dog & cat	Homemade	106	Brazil	To evaluate the estimated chemical composition and nutritional adequacy of home-prepared recipes published in Portuguese.

Table S2. Continuation.

Reference	Species	Diet category	n	Country	Study purpose
**Poblanno Silva et al., 2023 [90]**	Dog	Therapeutic (hepatic)	4	Canada	To evaluate dietary copper exposure in a dog with suspected copper-associated hepatitis and formulate a complete, balanced homemade diet with moderately low copper.
**Rowe et al., 2024 [91]**	Dog	Commercial & home-cooked	18	USA	Compares nutrient profiles of complete commercial and home-cooked diets and derives dietary targets including Na and Ca; whole-diet exposure is central to the study.
**Wilson et al., 2019 [92]**	Cat	Homemade maintenance	114	USA	To evaluate published home-prepared maintenance recipes for cats and compare software-derived nutrient profiles with NRC recommendations.

Table S3. Minimum and maximum recommended macro- and micromineral levels for dogs according to FEDIAF (2024) and AAFCO (2023) guidelines. For FEDIAF, recommended values are presented on a dry matter basis (per 100 g dry matter) and, in parentheses, on an energy basis (per 1000 kcal of metabolizable energy). AAFCO values are expressed only on a food-weight basis. Values are reported as minimum levels or minimum–maximum ranges. MER: maintenance energy requirement.

Mineral	Unit	FEDIAF				AAFCO	
		Adult MER (kcal/kg$^{0.75}$)		Early growth (< 14 weeks) & reproduction	Late growth (≥ 14 weeks)	Adult	Growth & reproduction
		95 kcal	110 kcal				
**Macrominerals**							
Calcium	g	0.58–2.50 (1.45–6.25)	0.50–2.50 (1.25–6.25)	1.00–1.60 (2.5–4.0)	0.80–1.80 [a] 1.00–1.80 [b] (2.0–4.5) [a] (2.5–4.5) [b]	0.5–2.5	1.2–2.5 1.2–1.8 [c]
Phosphorus	g	0.46–1.6 (1.16–4.0)	0.40–1.6 (1.0–4.0)	0.90 (2.25)	0.70 (1.75)	0.4–1.6	1.0–1.6
Ca:P ratio		1:1–2:1	1:1–2:1	1:1–1.6:1	1:1–1.8:1 [a] 1:1–1.6:1 [b]	1:1–2:1	1:1–2:1
Potassium	g	0.58 (1.45)	0.50 (1.25)	0.44 (1.10)	0.44 (1.10)	0.6	0.6
Sodium	g	0.12 (0.29)	0.10 (0.25)	0.22 (0.55)	0.22 (0.55)	0.08	0.3
Chloride	g	0.17 (0.43)	0.15 (0.38)	0.33 (0.83)	0.33 (0.83)	0.12	0.45
Magnesium	g	0.08 (0.20)	0.07 (0.18)	0.04 (0.10)	0.04 (0.10)	0.06	0.06
**Microminerals**							
Copper	mg	0.83–2.80 (2.08)	0.72–2.80 (1.80)	1.10–2.80 (2.75)	1.10–2.80 (2.75)	0.73	1.24
Iodine	mg	0.12–1.10 (0.30)	0.11–1.10 (0.26)	0.15–1.10 (0.38)	0.15–1.10 (0.38)	0.1–1.1	0.1–1.1
Iron	mg	4.17–68.18 (10.40)	3.60–68.18 (9.00)	8.80–68.18 (22.0)	8.80–68.18 (22.0)	4.0	8.8
Manganese	mg	0.67–17.0 (1.67)	0.58–17.0 (1.44)	0.56–17.0 (1.40)	0.56–17.0 (1.40)	0.50	0.72
Selenium (wet food)	µg	27.0–56.8 (67.5)	23.0–56.8 (57.5)	40.0–56.8 (100)	40.0–56.8 (100)	35–200	35–200
Selenium (dry food)	µg	22.0–56.8 (55.0)	18.0–56.8 (45.0)	40.0–56.8 (100)	40.0–56.8 (100)	35–200	35–200
Zinc	mg	8.34–22.7 (20.8)	7.20–22.7 (18.0)	10.0–22.7 (25.0)	10.0–22.7 (25.0)	8.0	10.0

[a] For puppies of dog breeds with adult body weight up to 15 kg, during the whole late growth phase (≥14 weeks); [b] for puppies of breeds with adult body weight over 15 kg, until the age of about 6 months. After that age, calcium can be reduced to 0.8 % dry matter (2 g/1000 kcal or 0.48 g/MJ) and the calcium-phosphorus ratio can be increased to 1.8/1; [c] the maximum of 1.8% is applicable to formulas that may be fed to large size puppies (those weighing 70 pounds or greater as mature lean adults). For other life stages, including non-large size growth formulas, the maximum calcium is 2.5% dry matter.

Table S4. Minimum and maximum recommended macro- and micromineral levels for cats according to FEDIAF (2024) and AAFCO (2023) guidelines. For FEDIAF, recommended values are presented on a dry matter basis (per 100 g dry matter) and, in parentheses, on an energy basis (per 1000 kcal of metabolizable energy). AAFCO values are expressed on a dry matter basis. Values are reported as minimum levels or minimum–maximum ranges. MER: maintenance energy requirement.

Mineral	Unit	FEDIAF			AAFCO	
		Adult MER (kcal/kg$^{0.67}$)		Growth & reproduction	Adult	Growth & reproduction
		75 kcal	100 kcal			
**Macrominerals**						
Calcium	g	0.53 (1.33)	0.40 (1.00)	1.00 (2.50)	0.6	1.0
Phosphorus	g	0.35 (0.85)	0.26 (0.64)	0.84 (2.10)	0.5	0.8
Ca:P ratio		1:1–2:1	1:1–2:1	1:1–1.5:1	1:1	1:1
Potassium	g	0.80 (2.00)	0.60 (1.50)	0.60 (1.50)	0.6	0.6
Sodium	g	0.10 (0.25)	0.08 (0.19)	0.16 (0.40)	0.2	0.2
Chloride	g	0.15 (0.39)	0.11 (0.29)	0.24 (0.60)	0.3	0.3
Magnesium	g	0.05 (0.13)	0.04 (0.10)	0.05 (0.13)	0.04	0.08
**Microminerals**						
Copper	mg	0.67–2.80 (1.67)	0.50–2.80 (1.25)	1.00–2.80 (2.50)	0.50	0.84 [a] 1.50 [b]
Iodine	mg	0.17–1.10 (0.43)	0.13–1.10 (0.33)	0.18–1.10 (0.45)	0.6	1.8
Iron	mg	10.70–68.18 (26.7)	8.00–68.18 (20.0)	8.00–68.18 (20.0)	8.0	8.0
Manganese	mg	0.67–17.00 (1.67)	0.50–17.00 (1.25)	1.00–17.00 (2.50)	0.76	0.76
Selenium (wet food)	µg	35.0–56.8 (87.5)	26.0–56.8 (65.0)	30.0–56.8 (75.0)	30	30
Selenium (dry food)	µg	28.0–56.8 (70.0)	21.0–56.8 (52.5)	30.0–56.8 (75.0)	30	30
Zinc	mg	10.0–22.7 (25.0)	7.5–22.7 (18.8)	7.5–22.7 (18.8)	7.5	7.5

[a] Copper in canned cat foods; [b] copper in extruded cat foods.

Table S5. Methodological and analytical quality assessment of studies reporting analytically measured mineral concentrations in complete dog and cat diets. The assessment comprised 11 criteria: c1, sampling strategy adequately described; c2, diet type, format, species, and relevant characteristics clearly described; c3, homogenisation procedure described; c4, digestion method described; c5, composite sampling from multiple packages described; c6, analytical method for mineral quantification clearly described and appropriate for the analytes measured; c7, analytical accuracy assessed using certified reference materials (CRM), recovery studies, or equivalent validation procedures; c8, duplicate or triplicate analyses performed; c9, limits of detection and/or quantification reported; c10, results reported with appropriate quantitative detail (individual values or a measure of variability); and c11, basis of expression of mineral concentrations clearly described (e.g., dry matter, as-fed, or energy basis). Each criterion was scored as 1 when fulfilled and 0 when not fulfilled or not reported. Scores are presented as descriptive indicators of methodological and analytical quality and should not be interpreted as formal estimates of risk of bias.

Reference	c1	c2	c3	c4	c5	c6	c7	c8	c9	c10	c11	Score
Abd-Elhakim et al., 2016 [17]	1	1	0	1	0	1	1	1	0	1	1	8
Akinrinmade & Akinrinde, 2011 [18]	1	1	0	0	1	0	0	0	0	1	1	5
Alborough et al., 2022 [19]	1	1	1	1	0	1	1	1	0	1	1	9
Altınok-Yipel et al., 2022 [20]	1	1	1	1	0	1	1	1	1	1	1	10
Alvarado et al., 2008 [21]	1	1	1	1	1	1	0	1	0	1	1	9
Atkins et al., 2011 [22]	1	1	1	1	0	1	0	0	0	1	1	7
Bilgiç et al., 2025 [23]	1	1	0	1	1	1	1	1	0	1	1	9
Bilgiç et al., 2026 [24]	1	1	0	1	1	1	0	1	0	1	1	8
Brociek et al., 2025 [25]	1	1	1	1	0	1	1	0	0	1	1	8
Brunetto et al., 2019 [26]	1	1	0	0	0	1	0	1	0	1	1	6
Cedeño-López & WingChing-Jones, 2022 [27]	1	1	1	1	0	1	0	0	0	1	0	6
Choi et al., 2023 [28]	1	1	0	1	0	1	0	1	0	1	1	7
Choi et al., 2024 [29]	1	0	1	1	0	1	1	1	1	1	0	8
Costa et al., 2018 [30]	1	1	1	1	0	1	1	1	1	1	0	9
Davies et al., 2017 [31]	1	1	1	1	0	1	1	1	1	1	1	10
De Nadai Fernandes et al., 2018 [32]	1	1	1	N/A	0	1	1	0	1	0	1	7
Dodd et al., 2021 [33]	1	1	1	1	0	1	0	0	0	1	1	7
Du et al., 2025 [34]	1	1	1	1	0	1	1	1	1	1	0	9
Dunham-Cheatham et al., 2021 [35]	1	1	1	1	0	1	1	1	0	1	1	9
Duran et al., 2010 [36]	1	1	0	1	0	1	1	1	0	1	1	8
Edinboro et al., 2013 [37]	1	1	0	1	0	1	0	0	1	1	1	7
Elias et al., 2012 [38]	1	1	1	N/A	0	1	1	0	0	0	1	6
Fantinati et al., 2021 [39]	1	1	0	0	0	1	0	0	0	1	1	5
Gagné et al., 2013 [40]	1	1	1	1	0	1	0	1	0	1	1	8
Guidi et al., 2021 [41]	1	1	0	0	0	1	0	1	1	1	1	7
Haydous et al., 2025 [42]	1	1	0	1	0	1	0	1	1	1	1	8
Ivars et al., 2016 [43]	1	1	1	1	1	1	0	0	0	1	1	8
Kalicharan et al., 2025 [44]	1	1	1	1	0	1	0	1	1	1	1	9
Kazimierska et al., 2020 [45]	1	1	1	1	1	1	0	1	0	1	1	9
Kim et al., 2018 [46]	1	1	1	1	0	1	0	1	0	1	1	8
Kim et al., 2018 [47]	1	1	1	1	0	1	0	1	1	1	1	9
Kępińska-Pacelik et al., 2023 [48]	1	1	1	1	0	1	1	1	0	1	1	9
Luippold et al., 2016 [49]	0	1	1	N/A	0	1	1	1	1	1	1	8
Luís et al., 2023 [50]	1	1	1	1	1	1	0	0	0	1	1	8
Macías-Montes et al., 2021 [51]	1	1	1	1	1	1	1	1	1	1	0	10

N/A indicates that c4 was not applicable because the analytical method did not require sample digestion.

Table S5. Continuation.

Reference	c1	c2	c3	c4	c5	c6	c7	c8	c9	c10	c11	Score
Mielczarek & Szydłowski, 2017 [52]	1	1	0	1	0	1	1	0	0	1	0	6
Naranjo-Jiménez et al., 2023 [53]	1	1	1	1	0	1	0	0	0	1	0	6
Olivindo et al., 2022 [54]	1	1	1	1	0	1	0	1	0	1	1	8
Or et al., 2024 [55]	1	1	0	1	0	1	0	1	0	1	1	7
Paulelli et al., 2018 [56]	1	1	1	1	0	1	1	1	1	1	0	9
Pedrinelli et al., 2021 [57]	1	1	1	1	0	1	0	1	0	1	1	8
Pereira et al., 2018 [58]	1	1	1	1	0	1	1	1	0	1	1	9
Prantil et al., 2016 [59]	1	1	1	0	0	1	0	0	0	1	1	6
Ryu et al., 2025 [60]	1	1	1	1	0	1	0	0	0	1	1	7
Sgorlon et al., 2022 [61]	1	1	1	1	1	1	1	1	0	1	1	10
Silva et al., 2021 [62]	1	1	1	1	0	1	1	1	1	1	1	10
Simcock et al., 2005 [63]	1	1	1	1	0	1	1	1	0	1	1	9
Sires et al., 2019 [64]	1	1	1	0	0	1	1	0	1	1	1	8
Squadrone et al., 2017 [65]	1	1	1	1	0	1	1	1	1	1	0	9
Summers et al., 2020 [66]	1	1	1	1	1	1	0	0	0	1	1	8
Summers et al., 2020 [67]	1	1	1	1	0	1	0	0	0	1	1	7
Summers et al., 2022 [68]	1	1	1	1	1	1	0	0	1	1	1	9
Tal et al., 2018 [69]	1	1	1	0	0	0	0	0	0	1	1	5
Taylor et al., 2009 [70]	1	1	0	0	0	0	0	0	0	1	1	4
Vecchiato et al., 2022 [71]	1	1	1	1	0	1	0	0	0	1	1	7
Vranić et al., 2023 [72]	1	1	0	1	0	1	0	1	0	1	0	6
Zafalon et al., 2020 [73]	1	1	1	1	0	1	0	1	0	1	1	8
Zafalon et al., 2021 [74]	1	1	1	1	0	1	0	1	1	1	1	9

N/A indicates that c4 was not applicable because the analytical method did not require sample digestion.
